# *Feroxichthys panzhouensis* sp. nov., a hump-backed colobodontid (Neopterygii, Actinopterygii) from the early Middle Triassic of Panzhou, Guizhou, China

**DOI:** 10.7717/peerj.11257

**Published:** 2021-04-07

**Authors:** Xin-Ying Ma, Guang-Hui Xu, Bing-He Geng

**Affiliations:** 1 Key Laboratory of Vertebrate Evolution and Human Origins of Chinese Academy of Sciences, Institute of Vertebrate Paleontology and Paleoanthropology, Chinese Academy of Sciences, Beijing, China; 2 CAS Center for Excellence in Life and Paleoenvironment, Beijing, China; 3 University of Chinese Academy of Sciences, Beijing, China

**Keywords:** Osteology, Phylogeny, Colobodontidae, Neopterygii

## Abstract

Neopterygii is a taxonomically diverse clade of ray-finned fishes, including Teleostei, Holostei and closely related fossil taxa. The Colobodontidae is a stem group of large-sized neopterygians with a durophagous feeding adaption from the Middle to Late Triassic marine ecosystems in Europe and South China. Here, we report the discovery of a new colobodontid, *Feroxichthys panzhouensis* sp. nov., based on a well-preserved specimen from the early Middle Triassic (Anisian) of Panzhou (formerly known as Panxian), Guizhou, China. The discovery extends the geographical distribution of *Feroxichthys* from eastern Yunnan into western Guizhou, and demonstrates a more rapid diversification of early colobodontids than previously thought. The new species possesses diagnostic features of *Feroxichthys* (e.g., a fused lacrimal-maxilla), but it is easily distinguished from the type species *Feroxichthys yunnanensis* and other colobodontids by some derived features on the skull and, especially, the relatively short and deep body with a prominent postcranial hump. This body form, previously unknown in colobodontids, implicates a morphological adaptation to structurally complex habitats in light of ecological studies of modern ray-finned fishes with a similar body form. In addition, the feeding apparatus suggests a more obligate durophagous diet for *F. panzhouensis* sp. nov. than other colobodontids. Results of a cladistic analysis recover the new species as a sister taxon of *F. yunnanensis* within the Colobodontidae, and suggest that a hump-backed body form has independently evolved multiple times in Triassic neopterygians. As such, the new finding provides an important addition for our understanding of the morphological and ecological diversity of neopterygian fishes from the Triassic marine ecosystems in South China.

## Introduction

Neopterygii is a taxonomically diverse clade of ray-finned fishes, including Teleostei (e.g., salmons and carps), Holostei (e.g., gars and bownfin), and closely related fossil taxa (Regan, 1923; Brough, 1931, 1939; Lehman, 1952; Schaeffer, 1956; Patterson, 1973, 1982; Gardiner, Maisey & Littlewood, 1996; Grande & Bemis, 1998; Coates, 1999; Arratia, 1999, 2013, 2015; Cavin, 2010; Grande, 2010; Sallan, 2014; Friedman, 2015; Xu & Zhao, 2016; Xu, 2019, 2021). This clade underwent a rapid radiation in the aftermath of the end-Permian extinction (Chen & Benton, 2012; Benton et al., 2013; Romano et al., 2016; Clarke & Friedman, 2018; López-Arbarello & Sferco, 2018; Romano, 2021). The Colobodontidae is a stem group of neopterygian fishes in the Middle to Late Triassic marine ecosystems (Stensiö, 1921; Bürgin, 1996; Mutter, 2002, 2004; Sun et al., 2008; Cartanyà et al., 2015; Li et al., 2019; Xu, 2020a). Members of this family are generally durophagous predators with a large body size (up to 650 mm in total length; Cartanyà et al., 2015), and consequently are important for understanding the trophic structure of Triassic marine ecosystems. Mutter (2002, 2004) restricted the family to include two genera *Colobodus* and *Crenilepis*; the former includes at least five species from the Middle to Late Triassic in Europe and South China, and the latter the type species *Crenilepis sandbergeri* from the Middle Triassic of Germany, Italy and Switzerland in Europe (Mutter, 2002; Sun et al., 2008; Cartanyà et al., 2015; Li et al., 2019). In addition, two new colobodontid taxa were proposed by Mutter (2002) in his doctoral dissertation but both have not been formally published yet. Recently, Xu (2020a) named a new colobodontid, *Feroxichthys yunnanensis* from the early Middle Triassic (Pelsonian, Anisian, ~244.2 Ma) of Luoping, Yunnan, China, which represents the earliest known member of this family (Zhang et al., 2009, 2015).

**Figure 1:**
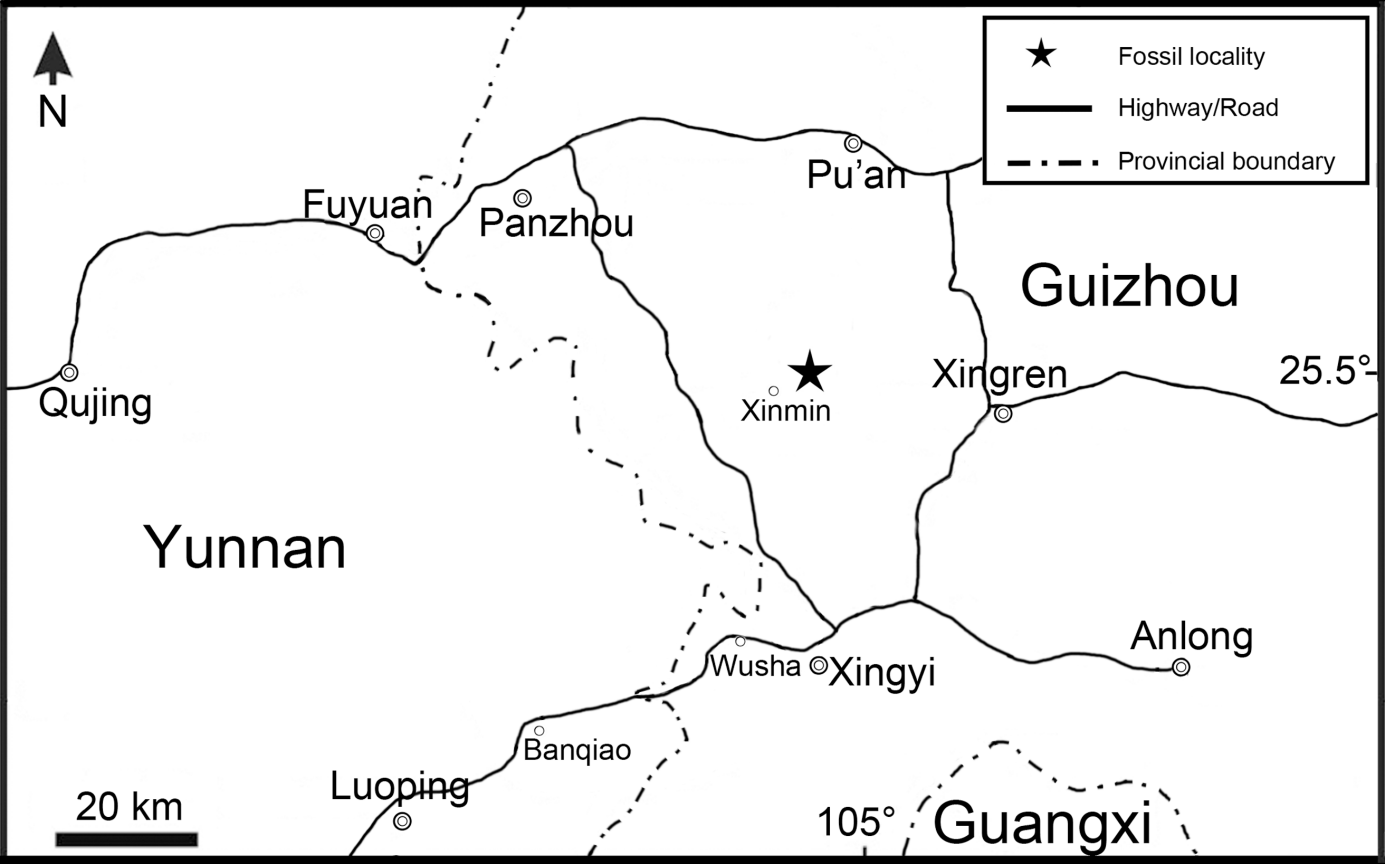
Fossil locality. Map showing the fossil locality of *Feroxichthys panzhouensis* sp. nov.

Here, we report the discovery of a new colobodontid fish on the basis of a large specimen collected in around 2005 by our colleague (Prof. F. Jin, retired last year) from Xinmin, Panzhou (formerly known as Panxian), Guizhou Province (Fig. 1). The specimen was embedded in gray muddy limestone from the upper part of the Second (Upper) Member of the Guanling Formation. Although some bones in the skull roof are missing, it is generally well-preserved, with a durophagous adaptation in feeding apparatus similar to other colobodontids. The new fish has a lacrimal fused with the maxilla, diagnostic of *Feroxichthys* (Xu, 2020a), but it bears a relatively short and deep body with a hump-backed postcranium, a feature previously unknown from the type species of *Feroxichthys* or other colobodontids. In the Middle Triassic, stem neopterygians with a similar or even deeper body form were previously known only by ‘perleidids’ or polzbergiids from the western Paleotethys Ocean in Europe (Tintori, 1990; Bürgin, 1992; Lombardo & Tintori, 2004). The new species of *Feroxichthys* presented in this contribution represents the first evidence of hump-backed stem neopterygian fishes from the eastern Paleotethys Ocean in Asia.

Along with the new species of *Feroxichthys*, other taxa from the same fossiliferous level in Panxian localities include invertebrates, marine reptiles, the colobodontid *Colobodus baii* (Sun et al., 2008), saurichthyiforms (Wu et al., 2011, 2013, 2015), holosteans (Chen et al., 2014; Xu & Shen, 2015) and birgeriiforms (Jiang et al., 2016). The whole fossil assemblage has been referred to the Panxian Fauna or Biota (Motani et al., 2008; Benton et al., 2013). Geological survey indicates that the Panxian Biota is slightly younger than the Luoping Biota because the former is recovered from the upper part of the Second Member of the Guanling Formation and the latter the middle part of this member of strata (Benton et al., 2013), although conodont analyses constrained both biotas within the same *Nicoraella kockeli* Zone (Sun et al., 2006, 2016; Zhang et al., 2009; Hu et al., 2011). The zircon dating provided an absolute age of 244.0 ± 1.3 Ma for the Panxian Biota (Wang et al., 2014).

## Materials and Methods

The specimen is curated at the fossil collections of the Institute of Vertebrate Paleontology and Paleoanthropology (IVPP), Chinese Academy of Sciences in Beijing, China. It was mechanically prepared with sharp steel needles and, for better contrast, was dusted with ammonium chloride (NH_4_Cl) before being photographed. The relative position of fins and scale counts were expressed following Westoll (1944). For ease of comparison with most existing literature, the traditional actinopterygian nomenclatures (e.g., Gardiner & Schaeffer, 1989; Bürgin, 1992; Grande & Bemis, 1998) are generally followed. However, the segmented and unbranched rays anterior to the principal rays of the fins are termed as procurrent rays rather than rudimentary rays, following Arratia (2008, 2009).

To illuminate the affinities of the new taxon, we incorporate it in a phylogenetic analysis based on the data matrix of Xu (2020a). The current data matrix includes 130 morphological characters and 56 actinopterygian taxa (see Supplemental Material). All characters were unordered and equally weighted. The basal actinopterygian *Moythomasia durgaringa* (Gardiner, 1984) was selected as the out-group taxon. The data matrix was generated by WinClada 1.00.08 (Nixon, 2002). Tree searches were accomplished with the heuristic search algorithm (gaps treated as missing data; 1,000 random addition sequence replicates; tree bisection-reconnection branch-swapping, with five trees held at each step and multiple trees saved) in PAUP* 4.0b10 (Swofford, 2003).

The electronic version of this article in Portable Document Format will represent a published work according to the International Commission on Zoological Nomenclature (ICZN), and hence the new names contained in the electronic version are effectively published under that Code from the electronic edition alone. This published work and the nomenclatural acts it contains have been registered in ZooBank, the online registration system for the ICZN. The ZooBank Life Science Identifiers can be resolved and the associated information viewed through any standard web browser by appending the LSID to the prefix http://zoobank.org/. The LSID for this publication is: urn:lsid:zoobank.org:pub: 03FCC5B3-E3E3-47B5-A0CE-7367BD37E64D. The online version of this work is archived and available from the following digital repositories: PeerJ, PubMed Central and CLOCKSS.

## Results

### Systematic paleontology

Actinopterygii Cope, 1887

Neopterygii Regan, 1923

Colobodontidae Andersson, 1916

*Feroxichthys* Xu, 2020a

*Feroxichthys panzhouensis* sp. nov.

**Figure 2:**
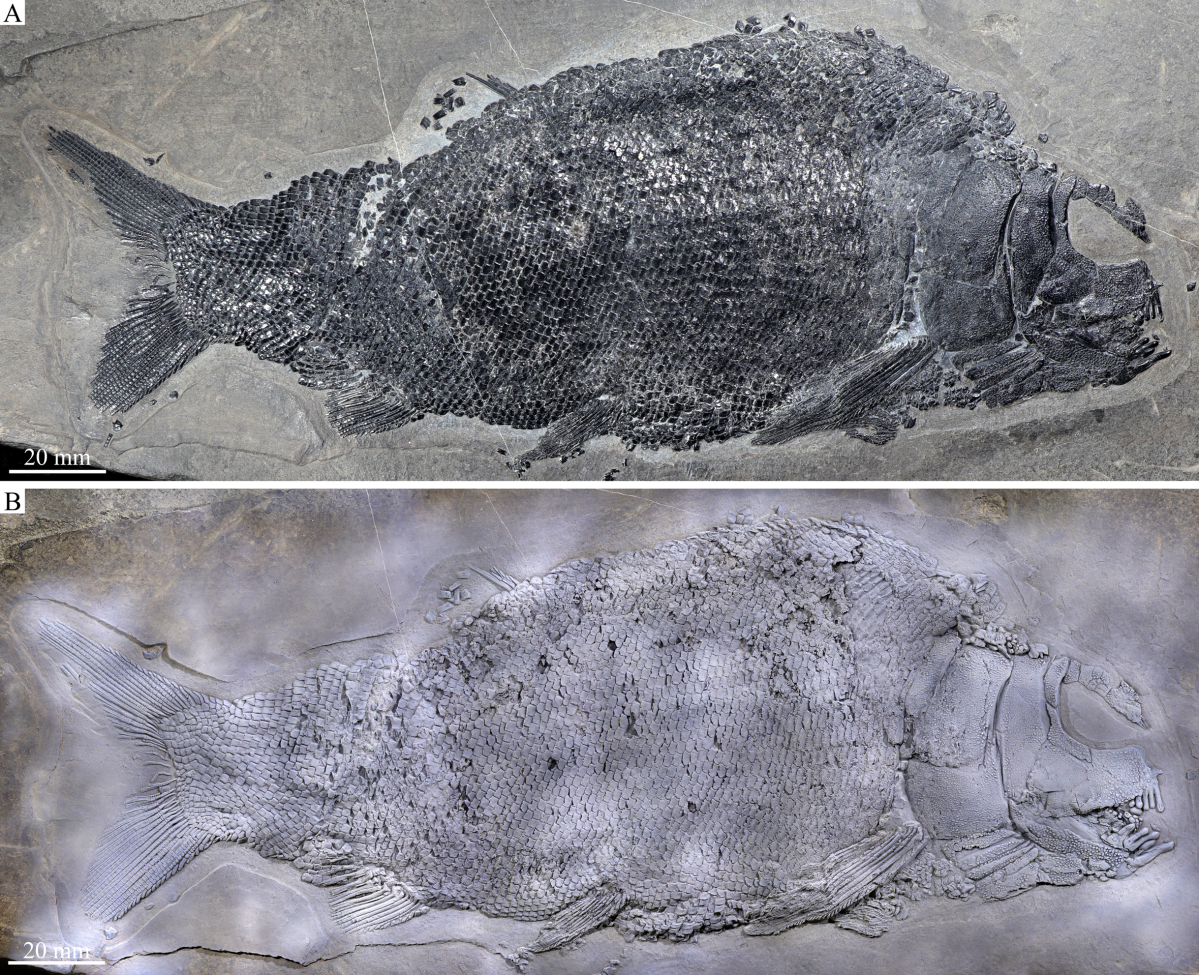
Entire specimen of the holotype. Entire specimen of *Feroxichthys panzhouensis* ****sp. nov., IVPP V16520 (holotype). (A) Original specimen. (B) Specimen dusted with ammonium chloride.

**Figure 3:**
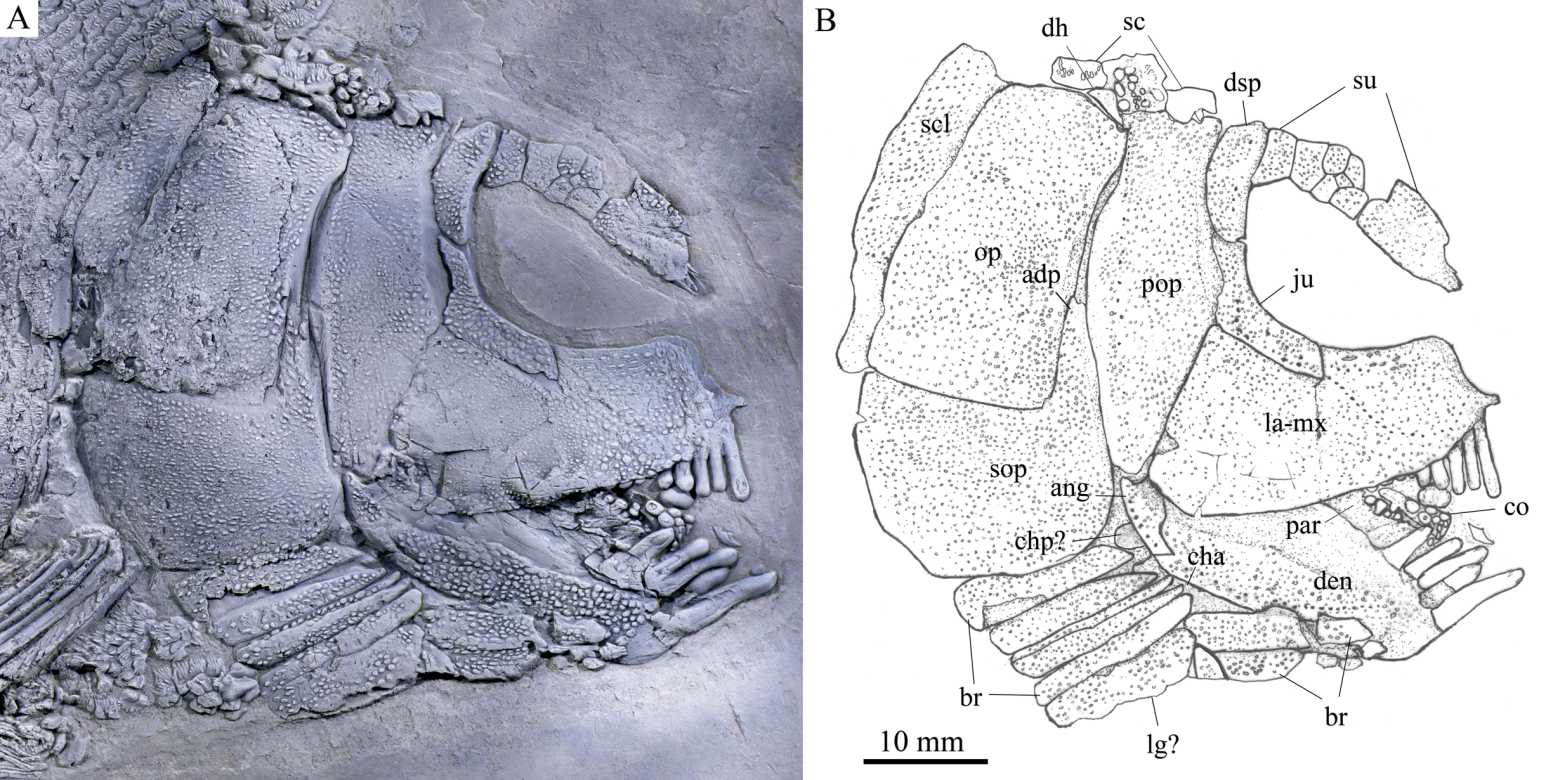
Skull and pectoral girdle of the holotype. Skull and pectoral girdle of *Feroxichthys panzhouensis* ****sp. nov., IVPP V16520 (holotype). (A) Photograph. (B) Line-drawing.

**Figure 4:**
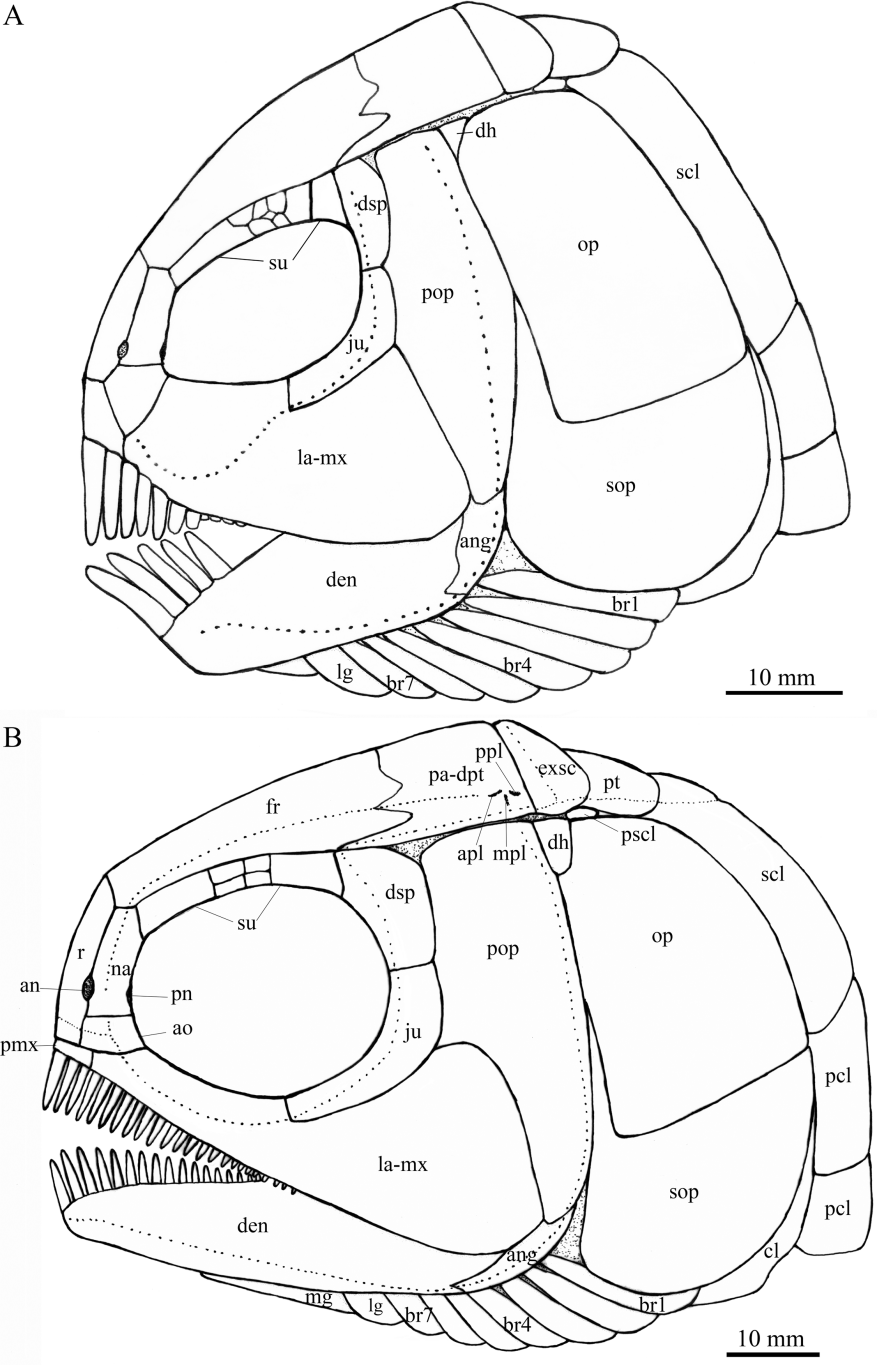
Reconstruction of skull and pectoral girdle. Reconstructions of skulls and pectoral girdles of two species of *Feroxichthys*. (A) *Feroxichthys* *panzhouensis* ****sp. nov.; the unlabeled bones are not preserved but tentatively recovered from the body plan of *F. yunnanensis*. (B) *Feroxichthys yunnanensis*.

**Figure 5:**
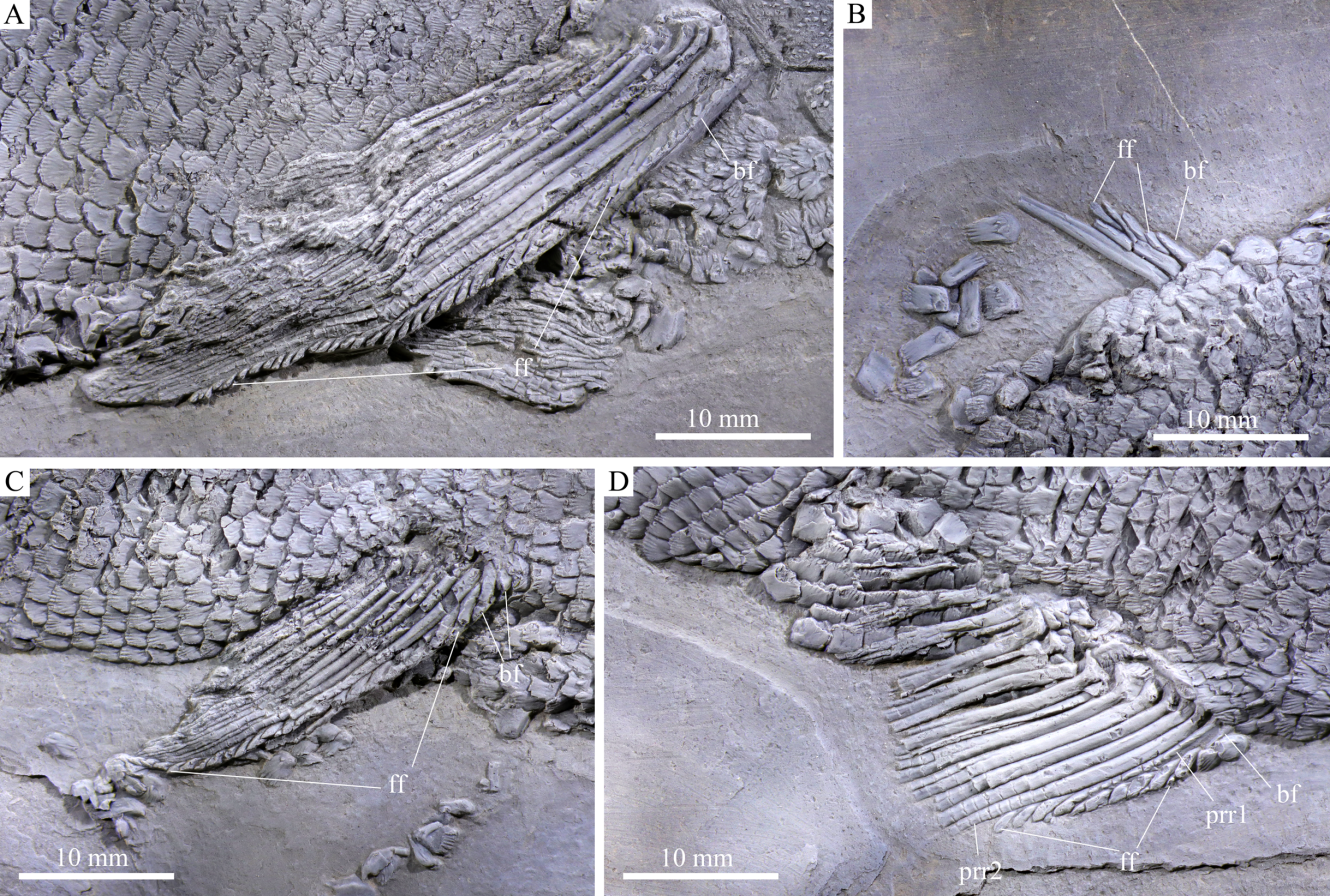
Fins. Fins of *Feroxichthys panzhouensis* ****sp. nov., IVPP V16520 (holotype). (A) Right pectoral fin, (B) dorsal fin, (C) right pelvic fin and (D) anal fin.

**Figure 6:**
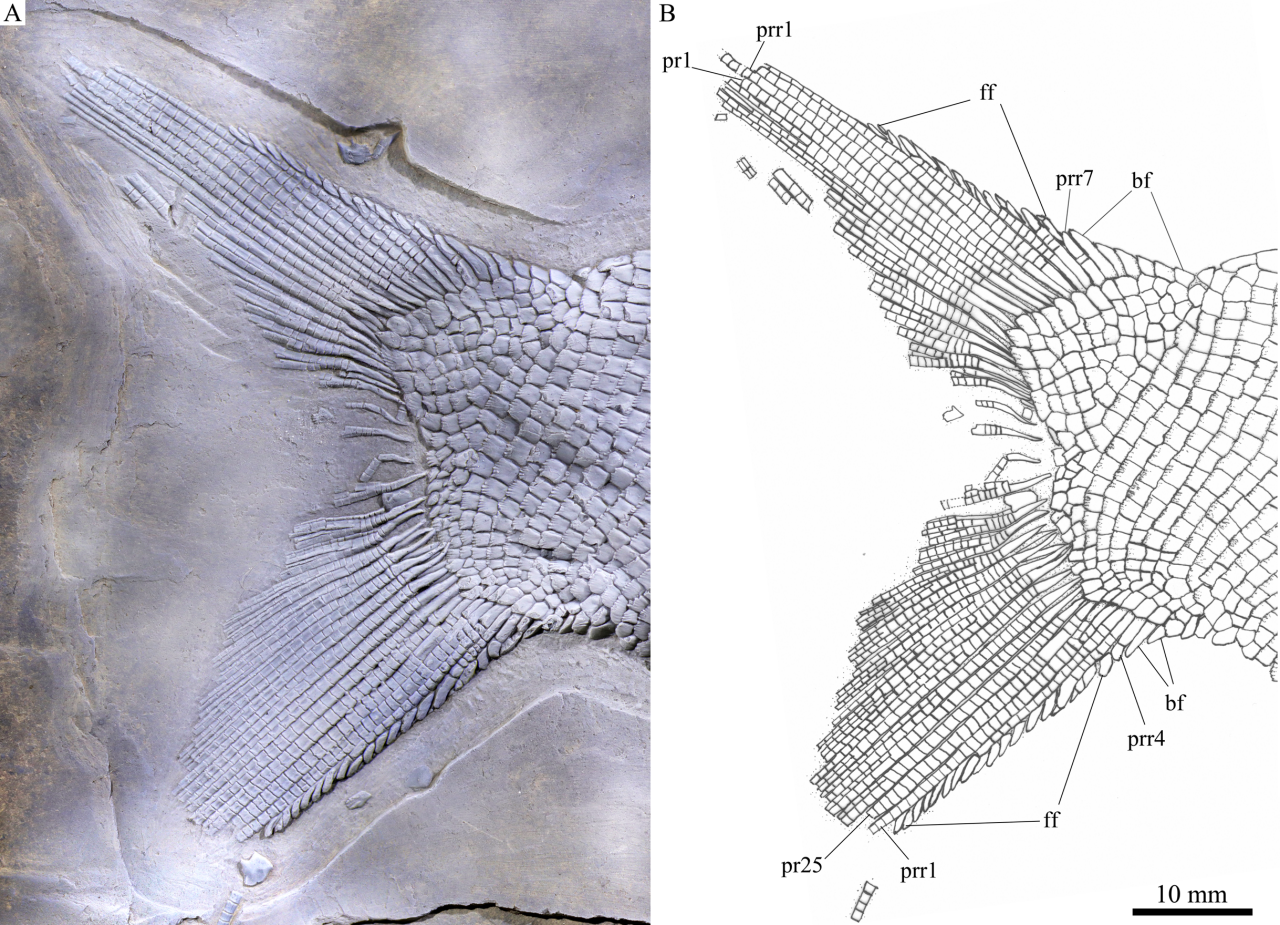
Caudal fin. Caudal fin of *Feroxichthys panzhouensis* ****sp. nov., IVPP V16520 (holotype). (A) Photograph. (B) Line-drawing.

**Figure 7:**
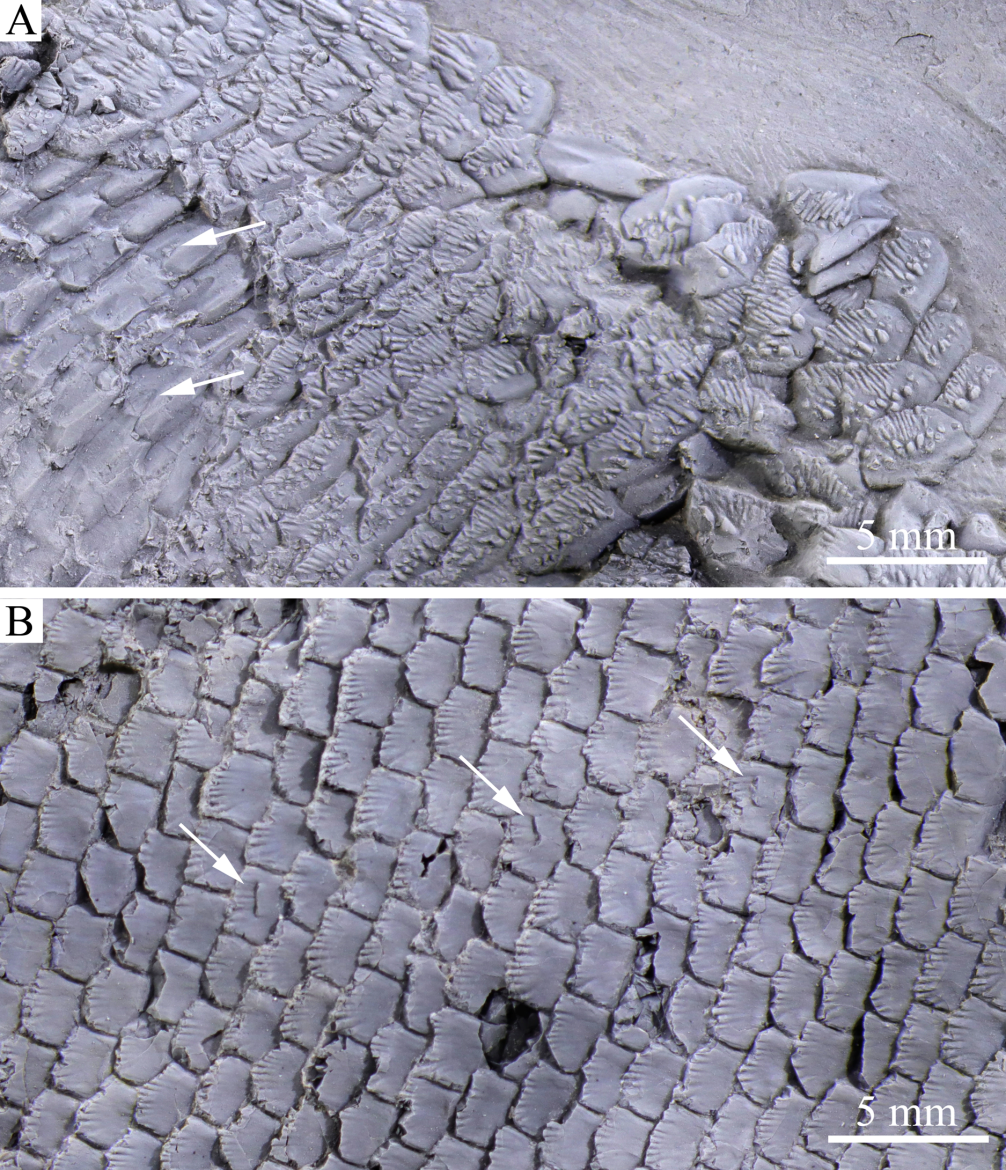
Scales. Scales of *Feroxichthys panzhouensis* ****sp. nov., IVPP V16520 (holotype). (A) Scales in predorsal region with arrows showing peg-socket articulations between scales. (B) Scales around the lateral line in middle flank region with arrows showing openings of pit organs.

**Figure 8:**
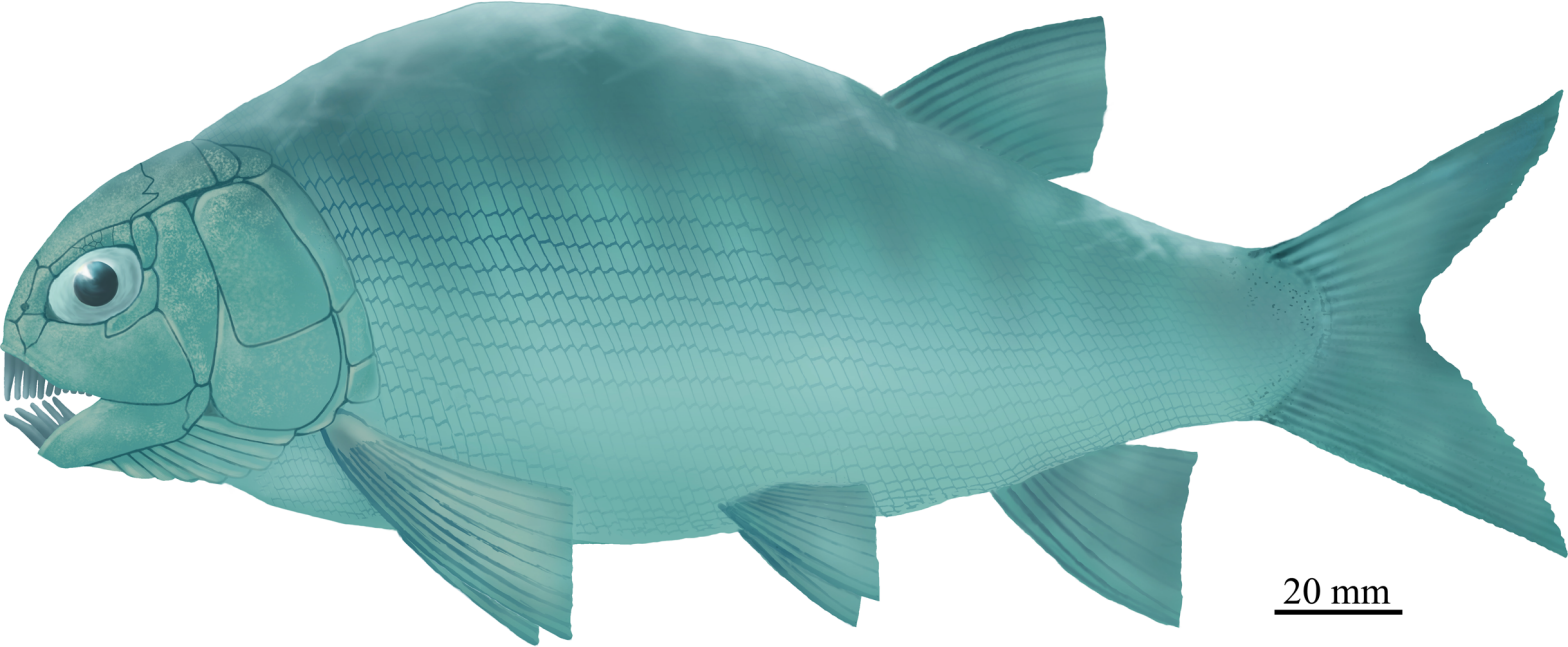
Life reconstruction. Life reconstruction of *Feroxichthys panzhouensis* ****sp. nov. (by Y. Xu and G.-H. Xu).

LSID urn:lsid:zoobank.org:act: 9D5DC42C-C6D1-4F14-817E-A54C4F43E78A

(Figures 2–3, 4A and 5–8)

**Etymology.** The specific epithet is derived from Panzhou, Guizhou Province, where the specimen was collected.

**Holotype.** IVPP V16520, a laterally compressed specimen with the skull roofing bones missing.

**Locality and horizon:** Xinmin, Panzhou, Guizhou; Second (Upper) Member of Guanling Formation, Pelsonian (244.0 ± 1.3 Ma; Wang et al., 2014), Anisian, Middle Triassic.

**Diagnosis:** A new species of *Feroxichthys* distinguished from the type species of the genus by the following features (autapomorphies, those unique among colobodontids, identified with an asterisk): relatively deep body with prominent postcranial hump (*); presence of nine supraorbitals (*); infraorbital portion of lacrimo-maxilla deeper than orbital radius (*); anterodorsal process of subopercle 38% of opercle in depth; opercle 1.6 times deeper than subopercle (excluding anterodorsal process); five long and strong, pencil-like teeth followed by four short and molariform teeth in anterior half of maxilla (*); five pencil-like teeth in dentary, slightly longer than those in maxilla (*); seven segmented procurrent rays in dorsal lobe of caudal fin; ornamentation of ridges and tubercles on anterior flank scales; and pterygial formula of D55/P25, A46, C77/T82 (*).

### Description

**General morphology and size.** *Feroxichthys panzhouensis* sp. nov. has a relatively deep body with a prominent postcranial hump and an abbreviated heterocercal caudal fin. The dorsal fin originates approximately midway between the origins of the pelvic and anal fins. The holotype (Fig. 2), the only known specimen, has a standard length of ~220 mm, a total length of ~260 mm, and a greatest body depth of 85 mm. The skull, strongly ornamented with dense ganoid tubercles and some striae (Fig. 3), has a length of 57 mm. The body is fully covered with rhombic scales, and because of this, the vertebral column and pterygiophores are not exposed (Fig. 4).

**Snout and skull roof.** The dermal bones in the snout region and skull roof are missing. They are tentatively reconstructed following the pattern of the type species of *Feroxichthys* (Fig. 4B).

**Circumorbital bones.** Eight trapezoidal or pentagonal supraorbitals are preserved in the holotype. In addition, there is a vacancy between the first (anteriormost) and other supraorbitals, which might represent a missing supraorbital. Thus, nine supraorbitals are recovered in this taxon (Fig. 4A). Among them, the first supraorbital is largest, being about half of the orbital length; the middle six are small, arranged in two or three lines; and the last two are relatively large, one-third as long as the first one.

The lacrimal has been fused with the infraorbital ramus of the maxilla, showing a characteristic feature of *Feroxichthys* among colobodontids. The infraorbital portion of the lacrimo-maxilla is significantly deeper than the orbital radius. The infraorbital sensory canal enclosed in the lacrimo-maxilla is curved; the anterior half of the canal is parallel to the oral margin of the maxilla, and the posterior half extends posterodorsally and enters the jugal from its anteroventral tip (Fig. 3).

The jugal is located at the posteroventral corner of the orbit. It is nearly L-shaped, having a nearly vertical dorsal arm and an anteriorly extended ventral ramus (Fig. 3). The infraorbital sensory canal traverses the jugal curvedly and passes onto the dermosphenotic dorsally.

The dermosphenotic is relatively narrow, twice as deep as the last supraorbital bone, with a slightly concave anterior margin and convex dorsal, ventral and posterior margins (Fig. 3). The dorsal half of the dermosphenotic anteriorly contacts the last supraorbital, and its ventral half forms the orbital margin. Posteriorly, the dermosphenotic contacts the preopercle directly, and a suborbital is evidently absent, as in other colobodontids.

**Jaws.** The premaxilla remains unknown because of incomplete preservation. As described above, the infraorbital ramus of the maxilla has fused with the lacrimal. The maxilla has a triangular posterior blade, which contacts the preopercle with a nearly straight posterodorsal margin (Fig. 3). The ventral (oral) margin of the maxilla is convex. The maximum depth of the posterior blade is as deep as the orbital length. Teeth are present only in its anterior half length of the oral margin, including five long and strong, pencil-like biting teeth followed by four stout crushing teeth. The first (anterior-most) tooth is the longest, and others gradually reduce in length posteriorly.

The lower jaw is robust and relatively deep, with two elements (dentary and angular) discernable in lateral view (Fig. 3). The supra-angular is unknown because of the lateral coverage of the maxilla. The dentary is the largest element of the lower jaw, having a nearly straight oral margin. The anterior and ventral parts of the dentary are ornamented with dense tubercles and its posterodorsal part is smooth. A row of five strong teeth is present in the oral margin of the dentary. They are pencil-like and slightly protruding, and their lengths are somewhat longer mor than those of anterior biting teeth in the maxilla. The distal ends of the teeth are blunt or slightly pointed rather than sharply pointed. The angular is small and roughly rhomboid in lateral view, contacting the dentary anteriorly. The mandibular sensory canal runs parallel to ventral margins of the angular and posterior portion of the dentary, and arches dorsally in the anterior portion of the latter bone.

An elongate coronoid and a plate-like prearticular are discernable near the oral margin of the dentary, and their oral margins are covered by rounded, molariform teeth with a prominent, wart-like knob on the tip (Fig. 3).

**Opercular series and dermohyal.** The preopercle is deep and vertically oriented (Fig. 3). It has a roughly trapezoidal dorsal part and a triangular ventral part that tapers ventrally. A vertical line of small pores near the posterior margin of the preopercle indicates the sensory canal in this bone.

The opercle is large and trapezoidal, having a depth/length ratio of 1.8. It has a slightly concave anterior margin and convex dorsal, ventral and posterior margins. The subopercle is sickle-shaped, bearing a deep anterodorsal process (Fig. 3). The process is 39% of the depth of the opercle. Excluding this process, the subopercle is 54% of the depth of the latter bone. In addition, a small triangular dermohyal is wedged between the preopercle and opercle.

**Branchiostegal rays, gulars and ceratohyals.** There are seven branchiostegal rays in right side of the skull (Fig. 3). They are relatively short and plate-like, tapering anteriorly. A relatively broad but shorter bone partly overlapping the branchiostegal rays is interpreted as a lateral gular because of its size and shape. The median gular, commonly present in other stem neopterygians, remains unknown because of incomplete preservation. The ceratohyals are partly exposed between the branchiostegal rays and lower jaw, including an elongate anterior ceratohyal and a subcircular posterior ceratohyal. No other bones of the hyoid arch are exposed.

**Paired girdles and fins.** Only the right supracleithrum is discernable in the pectoral girdle. It is relatively narrow and slightly anteriorly inclined, nearly as deep as the opercle.

The pectoral fins insert low on the body; 10 distally segmented and branched rays are discernable in the right pectoral fin (Fig. 6A). The first ray is the thickest and its proximal region is preceded by a basal fulcrum that is ornamented with elongate tubercles. The length of the basal fulcrum is about one-third of the length of the first ray. The second ray is longest, and others gradually reduce posteriorly in length. A series of leaf-like fringing fulcra is associated with the first ray, and their size gradually reduces posteriorly.

The pelvic girdles are not exposed. The pelvic fins insert at the 25th vertical scale row, each composed of ten distally segmented rays (Fig. 6C). The first ray is unbranched, preceded by two basal fulcra and a series of fringing fulcra; the remaining rays are branched distally.

**Median fins.** The dorsal fin originates above the 55th vertical scale row. Only anterior two rays are partly preserved in their proximal regions, preceded by a short basal fulcrum and several small fringing fulcra (Fig. 6B).

The anal fin originates below the 46th vertical scale row. It is composed of two procurrent rays and 11 principal rays (Fig. 6D). All rays are distally segmented; the anterior three rays are unbranched, and others branched distally. The first procurrent ray is no more than half of the length of the second one, preceded by a short basal fulcrum. A series of small fringing fulcra is associated with the leading margins of both procurrent rays. Notably, the distal segment of the first procurrent ray inserts between two fringing fulcra and resembles these fulcra in shape, showing a condition similar to that in *Colobodus baii* (Sun et al., 2008).

The caudal fin is abbreviated heterocercal with a forked profile (Fig. 7). It is composed of 25 principal rays, 12 in the dorsal lobe. Except two marginal principal rays, the middle rays are branched distally. Additionally, there are seven procurrent rays and six epaxial basal fulcra in the dorsal lobe, and four procurrent rays and two hypaxial basal fulcra in the ventral lobe. Rounded or elongated tubercles are discernable on the surfaces of some rays. Small, leaf-like fringing fulcra are associated with the leading margins of four procurrent rays (from the third to sixth) in the dorsal lobe and those of three procurrent rays (from the first to third) in the ventral lobe. The distal segments of these procurrent rays are similar to the fringing fulcra in shape, resembling the conditions in other colobodontids (Sun et al., 2008; Xu, 2020a).

**Scales.** The scales are rhomboid and presumedly ganoid with serrated posterior margins. They are arranged in 82 vertical rows between the pectoral girdle and the caudal inversion. In the 40th vertical row of scales, 18 and 19 scales are present above and below the lateral line on each side of the body, respectively. The scales are the largest in the anteroventral flank region with a depth/width ratio of 1.6, and they gradually become shorter dorsally, ventrally and posteriorly. The scales in the anterodorsal region of the body are ornamented with drop-like tubercles and parallel ridges or striae (Fig. 8A) and those in posterior regions are largely smooth except for some ridges extending anteriorly for a short length from the serrations at the posterior margin of the scale. The openings of lateral line system include vertical slits (openings of pit organs) in some lateral line scales (Fig. 8B); the slit is about 40% of the scale in depth. Peg-socket articulations are discernable from some scales in the anterodorsal flank region (Fig. 8A), as common for other early actinopterygians.

## Discussion

### Phylogenetic affinities and comparisons

**Figure 9:**
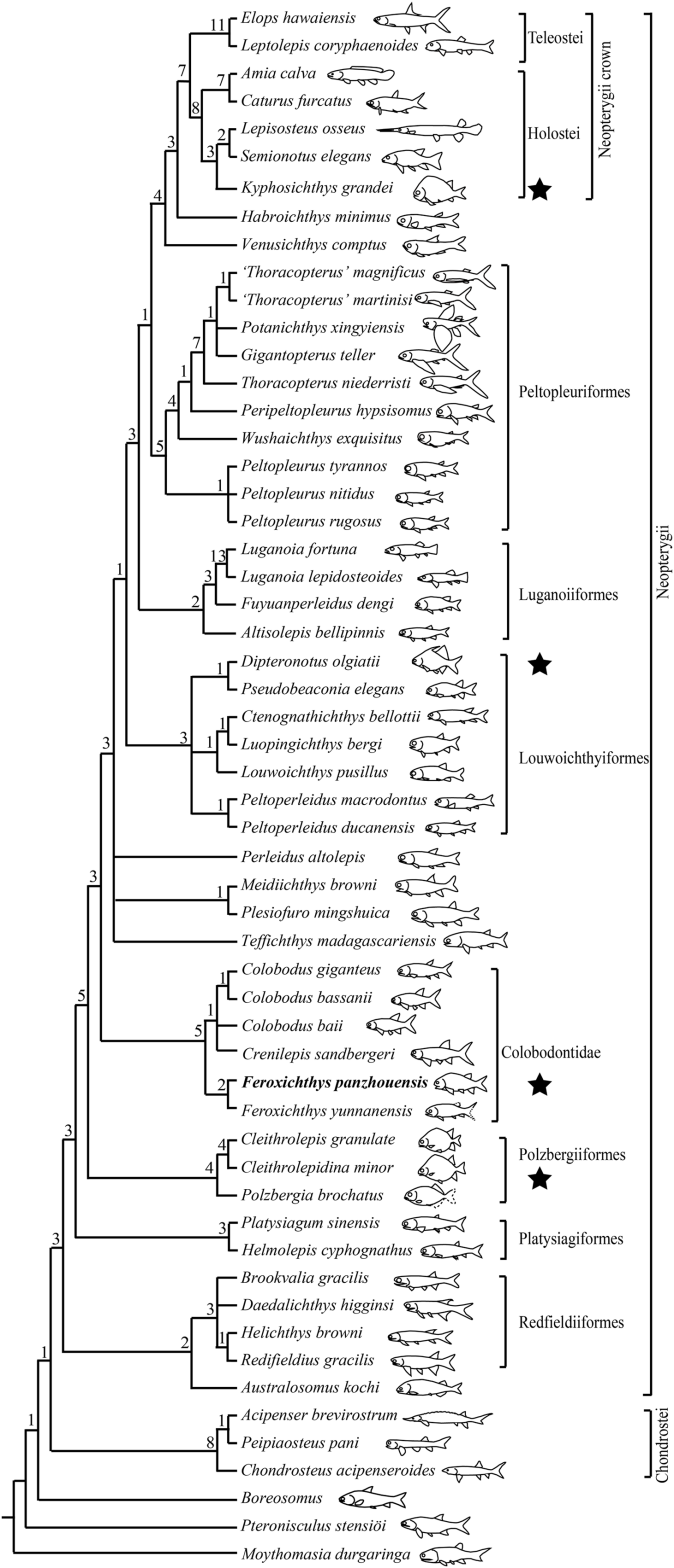
Strict consensus of most parsimonious trees. Strict consensus of 360 most parsimonious trees (tree length = 350 steps, consistency index = 0.4514, retention index = 0.7725), illustrating the phylogenetic position of *Feroxichthys panzhouensis* sp. nov. within the Neopterygii. Noticing that hump-backed body forms have independently evolved in polzbergiiform, colobodontid, pseudobeaconiid and kyphosichthyid neopterygians (marked with stars). Numbers above nodes indicate Bremer decay indices. For character descriptions and data matrix, see the online Supplemental Material.

The phylogenetic analysis resulted in 360 most parsimonious trees (length = 350 steps, consistency index = 0.4514, and retention index = 0.7725). The strict consensus tree (Fig. 9) is similar to that proposed by Xu (2020a), and *F. panzhouensis* sp. nov. is consistently recovered as a sister taxon to *F. yunnanensis* within the Colobodontidae. The nodes relevant to the studied new taxon are discussed below.

*Feroxichthys panzhouensis* sp. nov. is retrieved as a colobodontid because it possesses four synapomorphies of this family: presence of a deep anterodorsal process of the subopercle (independently evolved in holosteans); presence of multiple supraorbitals arranged in more than one horizontal rows (independently evolved in and *Caturus*; reversal in *Colobodus bassanii*); absence of suborbitals (independently evolved in platysiagiforms, *Pseudobeaconia*, *Habroichthys* and some crown neopterygians), and presence of ornamentation of round or elongated ganoid tubercles on caudal fin rays.

As in other colobodontids, *F. panzhouensis* sp. nov. lacks the derived features of *Teffichthys-*like taxa, *Perleidus altolepis* and more derived neopterygians: absence of the dermosphenotic/preopercle contact, presence of the opercle no larger than the subopercle (reversal in Peltopleuriformes and more derived neopterygians), no more than six pairs of branchiostegal rays (reversal in some crown neopterygians), and no more than 24 principal rays in the caudal fin (reversal in fuyuanperleidids and some louwoichthyids; Xu, 2021).

Within the Colobodontidae, the sister taxon relationships between *F. panzhouensis* sp. nov. and *F. yunnanensis* are supported by presence of a fused lacrimal-maxilla (independently evolved in luganoiiforms); distribution of teeth only in anterior half length of the maxilla (independently evolved in louwoichthyiforms and several other clades of early neopterygians); and presence of seven to nine pairs of branchiostegal rays (independently evolved in pholidopleuriforms and platysiagiforms). The genus *Feroxichthys* lacks the derived feature of other members (*Colobodus* and *Crenilepis*) of this family: presence of a postrostral (unknown in *F. panzhouensis* sp. nov. because of incomplete preservation; independently evolved in *Pseudobeaconia*; López-Arbarello & Zavattieri, 2008).

*Feroxichthys panzhouensis* sp. nov. is distinguished from other colobodontids by the following features (autapomorphies):

1. A prominent postcranial hump. *Feroxichtys panzhouensis* sp. nov. is unique among colobodontids in having a relatively deep and short body with a postcranial hump. By contrast, *F. yunnanensis* and other colobodontids generally have an elongated fusiform body.
2. Nine supraorbitals. *Feroxichthys panzhouensis* sp. nov. has a larger number (nine) of supraorbitals than *F. yunnanensis* (six) and many other colobodontids (three to five). Ten or more supraorbitals are known only in *Crenilepis* among colobodontids (Mutter, 2002).
3. Deeper infraorbital portion of lacrimo-maxilla. As described above, the infraorbital portion of the lacrimo-maxilla of *F. panzhouensis* sp. nov. is notably deeper than that of *F. yunnanensis*. Other colobodontids generally have a maxilla separated from the lacrimal. Outsides of colobodontids, a fused lacrimo-maxilla has independently evolved in luganoiiforms among early neopterygians (Bürgin, 1992; Xu, 2020a, 2020b).
4. Stronger but fewer teeth in dentary. The dentary of *F. panzhouensis* sp. nov. bear only five large teeth, showing the least number of teeth in this bone among colobodontids. These teeth are strong and pencil-like, enabling a powerful biting and a firmer grip in the prey capture. Similar teeth are otherwise present in *Ctenognatichys belotti* from the Middle Triassic of Switzerland and Italy (Bürgin, 1992; Bürgin & Herzog, 2002). By contrast, the teeth in the dentary are relatively slender, pointed and numerous in other colobodontids.
5. Short and stout crushing teeth in maxilla. Besides five long and pencil-like biting teeth, the maxilla of *F. panzhouensis* sp. nov. bear four short and stout crushing teeth. No other colobodontids have crushing teeth in the maxilla.
6. Larger number of procurrent rays in dorsal lobe of caudal fin. *F. panzhouensis* sp. nov. has seven segmented procurrent rays in the dorsal lobe of the caudal fin. This number is slightly less than that in *Crenilepis* and *Colobodus baii* (about eight) but is larger than those in *C. giganteus* and *C. bassanii* (two or three) and *F. yunnanensis* (four). Four or more epaxial procurrent rays are also present in the pholidopleuriforms, many redfieldiiforms, perleidids, derived louwoichthyids, luganoiiforms and most peltopleuriforms, but they are much reduced or lost in other early neopterygians (Lehman, 1952; Hutchinson, 1973; Griffith, 1977; Lombardo et al., 2011; Lin et al., 2011; Geng et al., 2012; Xu et al., 2012; Xu, Gao & Coates, 2015; Xu, Zhao & Shen, 2015; Xu, Ma & Zhao, 2018; Marramà et al., 2017; Wen et al., 2019).

### Implications

The new discovery extends the geographical distribution of the Ansian *Feroxichthys* from eastern Yunnan to western Guizhou, demonstrating a wider distribution for this genus. Previously, the earliest colobodontids were represented by *Colobodus baii* from the Panxian Biota (Sun et al., 2008) and *F. yunnanensis* from the Luoping Biota (Xu, 2020a) in South China. *Feroxichthys panzhouensis* sp. nov. documents the second colobodontid from the Panxian Biota, which is about two million years older than the European relatives near the Anisian/Ladinian boundary (~242 Ma) of the Besano Formation exposed in the Monte San Giorgio area in Europe (Bürgin, 1996; Mutter, 2002). The successive discoveries of two colobodontids in Yunnan and Guizhou indicate that the early diversification of this clade is more rapid than previously thought.

*Feroxichthys panzhouensis* sp. nov. represents the first colobodontid with a prominent postcranial hump. Modern ray-finned fishes with a similar body form (e.g., boarfish *Paristiopterus*) are commonly seen in structurally complex habitats, e.g., thick macrophyte beds, rocky areas or coral reefs; this body form provides an advantage for the overall force balance during swimming (Drucker & Lauder, 2002; Standen & Lauder, 2005; Helfman et al., 2009). In the Middle Triassic, a similar or even deeper body form were previously known by several polzbergiids and pseudobeaconiids in Europe (Tintori, 1990; Bürgin, 1992; Lombardo & Tintori, 2004; Lombardo, Rusconi & Tintori, 2008; Xu, 2021) and kyphosichthyid ginglymoidans in Asia (Xu & Wu, 2012; Xu, Ma & Zhao, 2018). As revealed by our analysis above, the colobodontid *Feroxichthys* is not closely related to these clades (Fig. 9), and this implicates that a hump-backed body form has independently evolved multiple times in Triassic neopterygians.

The feeding apparatus suggests a more obligate durophagous diet for *F. panzhouensis* sp. nov. than other colobodontids. It is generally accepted that colobodontids evolved a durophagous diet with dentition combining grasping and crushing morphologies. Similar dentition is present in living durophagous fishes (e.g., the cichlid *Astatoreochromis alluaudi*; Helfman et al., 2009), which employ anterior peg-like or conical teeth for initial prey capture and flatted or rounded molariform teeth for some sort of crushing in the oral cavity. *Feroxichthys panzhouensis* sp. nov., similar to other colobodontids, have molariform teeth on the coronoids, prearticular and pterygoids. Moreover, it has blunted, crushing teeth on the maxilla. These teeth could be important in the initial crushing on the hard-shelled during the prey capture. In addition, *F. panzhouensis* sp. nov. probably has a slower swimming ability than other colobodontids because its hump-backed body form causes larger drags during swimming. Accordingly, its prey is likely slow-moving. The potential invertebrate prey of *F. panzhouensis* sp. nov. would be gastropods, brachiopods and bivalves in the same ecosystems.

## Conclusion

Our study of a large well-preserved specimen from the Middle Triassic (Anisian) Panxian Biota recovers the first colobodontid fish with a prominent postcranial hump. The new colobodontid possesses diagnostic features of *Feroxichthys* but is easily distinguished from other members of this family by some autapomorphies on skull and body form. Results of a phylogenetic analysis recovers it as a sister taxon of *F. yunnanensis* and provides insights into the interrelationships of early neopterygians. The teeth of *F. panzhouensis* sp. nov. are well adapted for a durophagous diet, and its potential prey would be mainly hard-shelled, slow-moving invertebrates, e.g., gastropods, brachiopods and bivalves. The discovery provides an important addition to our understanding of the early diversification of colobodontids, and more generally, the morphological and ecological diversity of early neopterygians from the early Middle Triassic marine ecosystems in South China.

## Supplemental Information

10.7717/peerj.11257/supp-1 Supplemental Information 1 Anatomical character list, data matrix, and the strict consensus tree showing all character optimizations.

10.7717/peerj.11257/supp-2 Supplemental Information 2 Data matrix.

## Anatomical Abbreviations

adp: anterodorsal process of subopercle
an: anterior nostril
ang: angular bone
ao: antorbital bone
apl: anterior pit-line
bf: basal fulcrum
br: branchiostegal rays
cha: anterior ceratohyal
chp: posterior ceratohyal
cl: cleithrum
co: coronoid
den: dentary
dh: dermohyal
dsp: dermosphenotic
exsc: extrascapular bone
ff: fringing fulcrum
fr: frontal bone
la-mx: lacrimo-maxilla
lg: lateral gular
ju: jugal
mg: median gular bone
mpl: middle pit-line
na: nasal bone
op: opercle
pa-dpt: parieto-dermopterotic
par: prearticular bone
pcl: postcleithrum
pmx: premaxilla
pn: posterior nostril
pop: preopercle
ppl: posterior pit-line
ppr: procurrent ray
pr: principal ray
pscl: presupracleithrum
pt: posttemporal
r: rostral bone
sc: scale
scl: supracleithrum
sop: subopercle
su: supraorbital bone

## Additional Information and Declarations

### Data Availability

The following information was supplied regarding data availability: The online Supplemental Material are available in the Supplemental Files at PeerJ, including the anatomical character list, data matrix, and the strict consensus tree showing all character optimizations. The specimen (IVPP V16520) illustrated and described is stored at the fossil collections of the Institute of Vertebrate Paleontology and Paleoanthropology, Chinese Academy of Sciences in Beijing, China.

### New Species Registration

The following information was supplied regarding the registration of a newly described species: Publication LSID: urn:lsid:zoobank.org:pub:03FCC5B3-E3E3-47B5-A0CE-7367BD37E64D *Feroxichthys panzhouensis* sp. nov LSID: urn:lsid:zoobank.org:act: 9D5DC42C-C6D1-4F14-817E-A54C4F43E78A
