# Supplementary material for: Feroxichthys panzhouensis sp. nov., a hump-backed colobodontid (Neopterygii, Actinopterygii) from the early Middle Triassic of Panzhou, Guizhou, China
Source: PeerJ. 2021 Apr 7;9:e11257. doi: 10.7717/peerj.11257 (PMC8035898; doi:10.7717/peerj.11257)
Supplement: Supplemental Information 1 [file peerj-09-11257-s001.pdf]

Online Supplementary Material

Feroxichthys panzhouensis sp. nov., a hump-backed colobodontid (Neopterygii, Actinopterygii) from the early Middle Triassic of Panzhou, Guizhou, China

Table of Contents

1. Taxa and principal sources of data
2. Supplementary figure
3. Character list
4. Data matrix
5. References to supplementary information

1. Taxa and principal sources of data

FMNH, Field Museum of Natural History, Chicago, USA

IVPP, Institute of Vertebrate Paleontology and Paleoanthropology, Chinese Academy of Sciences, Beijing, China

NHMUK, Natural History Museum, London, UK;

PIMUZ, Paläontologisches Institut und Museum, Universität Zürich, Zürich, Switzerland

Moythomasia durgaringa: Gardiner, 1984

Australosomus kochi: Nielsen, 1949; NHMUK P17141–17143, 17156, 17157, 17160, 17161, 20940–20945

Acipenser brevirostrum: Hilton et al., 2011

Amia calva: Grande and Bemis, 1998

Boreosomus piveteaui: Nielsen, 1942

Brookvalia gracilis: Hutchinson, 1973b

Caturus furcatus: FMNH UC2057; Patterson, 1975; Grande and Bemis, 1998

Chondrosteus acipenseroides: Hilton and Forey, 2009

Cleithrolepidina minor: Hutchinson, 1973b

Cleithrolepis granulate: Wade, 1935; Hutchinson, 1973b

Colobodus baii: Sun et al., 2008; IVPP V19974

Colobodus bassanii: Mutter, 2002, 2004

Colobodus giganteus: Cartanyà et al., 2015

Crenilepis sandbergeri: Mutter, 2002, 2004

Ctenognathichthys bellottii: Bürgin, 1992

Daedalichthys higginsi: Hutchinson, 1973b

Dipteronotus olgiatii: Tintori, 1990

Elops hawaiensis: Forey, 1973

Feroxichthys yunnanensis Xu, 2020

Fuyuanperleidus dengi: Geng et al., 2012; Sun et al., 2012

Gigantopterus teller: Griffith, 1977

Habroichthys minmius: Bürgin, 1992; PIMUZ T196, 233, 2917, 2883, 2884

Helichthys browni: Hutchinson, 1973b

Helmolepis cyphognathus: Neuman & Mutter, 2005

Kyphosichthys grandei: Xu & Wu, 2012

Lepisosteus osseus: Grande, 2010

Leptolepis coryphaenoides: Patterson, 1975; Arratia, 1999, 2013

Luganoia lepidosteoides: Bürgin, 1992

Luganoia fortuna: Xu, 2020b

Luopingichthys bergi: Sun et al., 2009

Louwoichthys pusillus: Xu, 2020a

Meidiichthys browni: Brough, 1931; Hutchinson, 1973b

Perleidus altolepis: Lombardo, 2001

Peipiaosteus pani: Zhou, 1992

Peltopleurus rugosus: Bürgin, 1992; PIMUZ T2904

Peltopleurus nitidus: Xu and Zhao, 2016

Peltopleurus tyrannos: Xu et al., 2018

Peltoperleidus ducanensis: Bürgin et al., 1991

Peltoperleidus macrodontus: Bürgin, 1992

Peripeltopleurus hypsisomus: Bürgin, 1992; PIMUZ T1211, 2150, 2869

Pseudobeaconia elegans: Hutchinson, 1973a; López-Arbarello and Zavattieri, 2008

Platysiagum sinensis: Wen et al., 2019

Plesiofuro mingshuica: Xu et al., 2015a

Polzbergia brochatus: Griffith, 1977

Potanichthys xingyiensis: Xu et al., 2012

Pteronisculus stensioi: Nielsen, 1942; NHMUK P16282, 16283, 16300, 163001, 16307–16308

Redifieldius gracilis: Schaeffer and McDonald, 1978

Semionotus elegans: Olsen and McCune, 1991; Cavin, 2010; Grande, 2010; López-Arbarello, 2012

Teffichthys (*'Perleidus'*) *madagascariensis*: NHMUK P16247, 16248, 19580–19584, 19587–19592, 19595–19599, 19603–19620, 19622, 19623; Lehman, 1952; Patterson, 1975; Marramà et al., 2017

Thoracopterus niederristi: Griffith, 1977; Lehman, 1979; NHMUK P1098

'Thoracopterus' magnificus: Tintori and Sassi, 1992

'Thoracopterus' martinisi: Tintori and Sassi, 1992

Venusichthys comptus: Xu and Zhao, 2016

Wushaichthys exquisitus: Xu et al., 2015b

2. Supplementary Figure

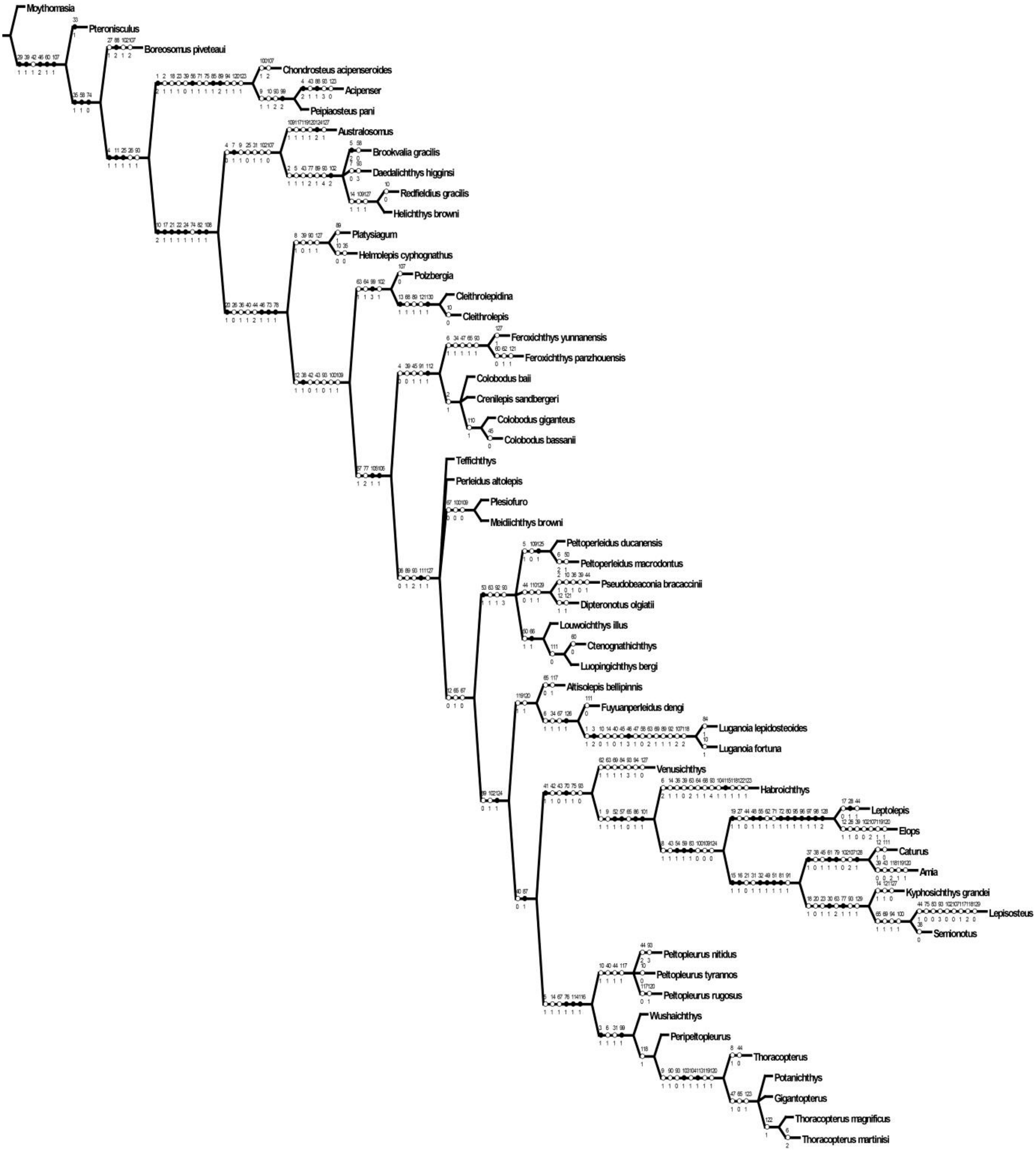

Supplementary Figure S1. Strict consensus of 360 most parsimonious trees (tree length = 350 steps, consistency index = 0.4514, retention index = 0.7725), illustrating the phylogenetic position of *Feroxichthys panzhouensis* sp. nov. within the Neopterygii. Character changes indicated with solid circles are unique.

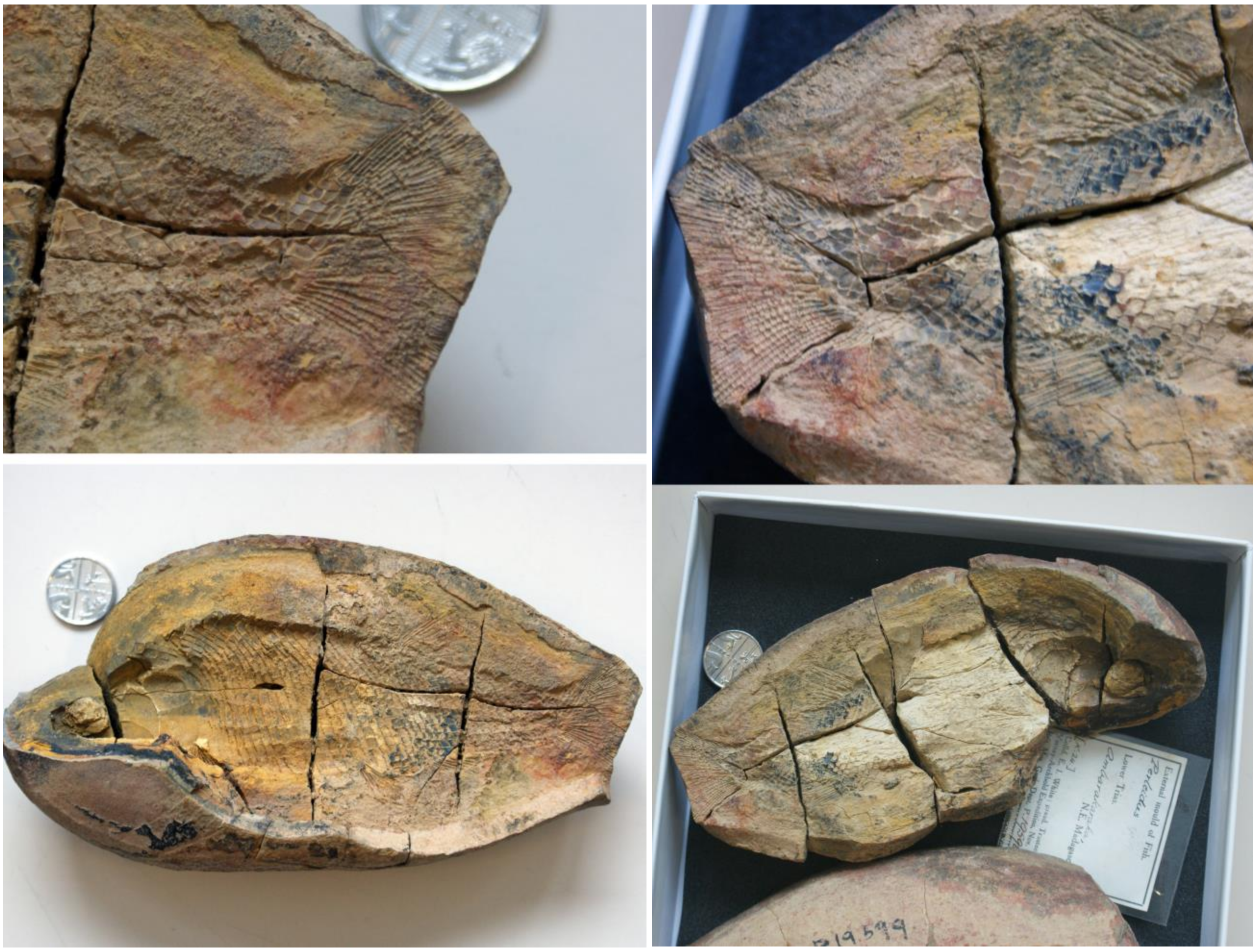

Supplementary Figure S2. *Teffichthys* (*'Perleidus'*) *madagascariensis* from the Lower Triassic of Madagascar. NHMUK P19599. Notably, three epaxial procurrent rays are present in the dorsal lobe of the caudal fin (contra the description of Marramà et al., 2017).

3. Character list

The characters are mainly adopted or modified from previous publications on the phylogenetic relationships of the Neopterygii (A, Arratia, 2013; C, Coates, 1999; CA, Cloutier and Arratia, 2004; GS, Gardiner and Schaeffer, 1989; GML, Gardiner, Maisey and Littlewood, 1996; GSM, Gardiner, Schaeffer and Masserie, 2005); GB, Grande and Bemis, 1998; G, Grande, 2010; LZ, López-Arbarello and Zavattieri, 2008; L, López-Arbarello, 2012; P, Pinna, 1996; XG, Xu and Gao, 2011; XW, Xu and Wu, 2012; X, Xu et al., 2012; XGF, Xu, Gao and Finarelli, 2014; XZS, Xu, Zhao and Shen, 2015b; XGC, Xu, Gao and Coates, 2015a; XZ, Xu and Zhao, 2016; XM, Xu and Ma, 2016; XMZ, Xu, Ma and Zhao, 2018; XA, Xu, 2020a).

Skull Roof

(1) Rostral: large, shield-like or cap-like (0); small, much reduced or lost by fusion with other elements (1); irregularly shaped and anamestic (2). (Modified from GML19; XW17; XGC51; XM1; XMZ1; XA1)

(2) Postrostral: absent (0); present (1). (XA2)

(3) Frontal(s): elongate (0); laterally expanded (1); well constricted above orbit (2). (X33; XGC6; XM3; XMZ3; XA3)

(4) Contact relationships of frontals: anterior portions of frontals partly separated by median rostral bone (0); fully contact each other medially or fused into a median element (1); paired, completely separated by rostral bones (2).

(5) Supraorbital sensory canal: ending in parietal (0); ending in frontal (1); ending in dermopterotic (2). (Modified from X34; XGC7; XZS34; XM4; XMZ4; XA4)

(6) Distinct parietal: present (0); absent, fused with dermopterotic (1); absent, fused with frontal and dermopterotic (2). (Modified from XZS28; XM5; XMZ5; XA5)

Remarks: Based on personal observation of the holotype, the parietal is fused with the dermopterotic in *Fuyuanperleidus dengi* from the Middle Triassic (Anisian) of Luoping, Yunnan.

(7) Number of parietals: one pair (0); three or more (1). (Modified from XA6)

(8) Nasal bones: completely separated from each other by median rostral or rostral bones (0); joined or nearly joined in midline (1). (XM6; XMZ6; XA7)

(9) Nasal bone forming part of orbital margin: present (0); absent (1). (XZ55; XM7; XMZ7; XA8)

(10) Number of extrascapulars: two pairs or more (0); three (1); one pair (2). (Modified from X16; XGC8;

XM23; XMZ23; XA9)

(11) Intertemporal: present (0); absent (1). (Modified from GSM17; XGC10; XM24; XMZ24; XA10)

(12) Ratio of dermopterotic length to parietal length: less than two (0); two or more (1).

(13) Accessory dermopterotic: absent (0); present (1). (XGC59; XM25; XMZ25; XA11)

(14) Dermopterotic/preopercle contact: present (0); absent (1). (XM26; XMZ26; XA12)

Neurocranium

(15) Sphenotic with small dermal component: absent (0); present (1). (G23; XW16; XM8; XMZ8; XA13)

(16) Pterotic: present (0); absent (1). (GML2; GMC47; XM9; XMZ9; XA14)

(17) Opisthotic: present (0); absent (1). (G33; XGC48; XM10; XMZ10; XA15)

(18) Intercalar: present (0); absent (1). (GML4; XM11; XMZ11; XA16)

(19) Supraoccipital: absent (0); present (1). (G28; XGC49; XM12; XMZ12; XA17)

(20) Post-temporal fossa: absent (0); present (1). (GS28; XGC1; XM13; XMZ13; XA18)

(21) Sub-temporal fossa: absent (0); present (1). (GS29; XGC2; XM24; XMZ14; XA19)

(22) Dilatator fossa: absent (0); present (1). (GS31; XGC3; XM15; XMZ15; XA20)

(23) Posterior myodome: present (0); absent (1). (Modified from GS10; GSM6; XM16; XMZ16; XA14; XA21)

(24) Anterodorsal myodome: present (0); absent (1). (XGF8; XZ8; XM17; XMZ17; XA14; XA22)

(25) Parasphenoid: short, terminates at otic fissure (0); long, extends across otic fissure (1). (Modified from GSM8; XGC4; XM18; XMZ18; XA23)

(26) Basipterygoid process: present (0); absent (1). (GSM12; XGC5; XM19; XMZ19; XA24)

(27) Internal carotid foramen on parasphenoid: absent (0); present (1). (GML14; XGC53; XM20; XMZ20; XA25)

(28) Efferent pseudobranchial foramen on parasphenoid: absent (0); present (1). (GML15; XGC54; XM21; XMZ21; XA26)

(29) Pineal foramen: present (0); absent (1). (XGF113; XA27)

Circumorbital Bones

(30) Anterior infraorbital bone(s): absent (0); present (1). (Modified from G21; XW24; XGC40; XM27; XMZ27; XA28)

(31) Antorbital (when it is not fused with premaxilla): small, shorter than nasal (0); enlarged, nearly equal to or deeper than nasal (1). (Modified from XA29)

(32) Tube-like canal bearing anterior arm of antorbital: absent (0); present (1). (G12; XW19; XGC43; XM28; XMZ28; XA30)

(33) Anterior part of lacrimal bearing teeth: absent (0); present (1). (Modified from XA31)

(34) Lacrimal: independent (0); fused with maxilla (1).

Remarks: Based on personal observation on the holotype, the lacrimal is fused with the maxilla in *Fuyuanperleidus dengi* from the Middle Triassic (Anisian) of Luoping, Yunnan.

(35) Dermosphenotic/nasal contact: present (0); absent (1). (Modified from GS19; XGC11; XM29; XMZ29; XA32)

Remarks: In *Helmolepis cyphognathus*, the triangular ‘supraorbital’ is better interpreted as a dermosphenotic according to its shape and position (Neuman & Mutter, 2005).

(36) Dermosphenotic/preopercle contact: absent (0); present (1). (XA33)

(37) Dermosphenotic attachment to skull roof in adult-sized individuals: loosely attached on the skull roof or hinged to the side of skull roof (0); firmly sutured into skull roof, forming part of it (1). (GB56; XGC13; XM31; XMZ31; XA34)

(38) Position of dermosphenotic relative to dermopterotic (intertemporal plus supratemporal): located at nearly same horizontal level of dermopterotic (0); located below dermopterotic (1).

(39) Suborbital(s): absent (0); present (1). (Modified from GS9; XM35; XMZ35; XA35)

Remarks: The coding (1) for *Australosomus* is based on personal observation on NHMUK 17157.

(40) Positions of suborbital(s): extending below dermosphenotic (0); located posterior to dermosphenotic only (1). (Modified from XA36 and 37)

(41) Number of infraorbitals between antorbital and dermosphenotic: three or less (0); four or more (1). (Modified from GS21; XGC16; XM33; XMZ33; XA38)

(42) Postinfraorbital(s): absent (0); present (1). (XA39)

(43) Supraorbital: absent (0); present (1). (Modified from GS14; XGC17; XM34; XMZ34; XA40)

(44) Number of supraorbital bones: single (0); two (1); three or more (2). (Modified from GS14; XGC17; XM34; XMZ34; XA41)

Remarks: Based on personal observation, four supraorbital bones are present in *Luopingichthys bergi* from the Middle Triassic (Anisian) of Luoping, Yunnan.

(45) Multiple supraorbital bones arranged in more than one horizontal rows: absent (0); present (1).

Jaws and dentation

(46) Premaxilla: fused with antorbital, bearing sensory canal (0); present as distinct elements, lacking sensory canal (1); lost (2); fused with rostral, bearing sensory canal (3). (Modified from X30; XM36; XMZ36; XA42)

Remarks: Personal reexaminations on the type specimens confirm that a pair of small, toothed premaxillae lacking sensory canal is present in *Platysiagum sinensis*.

(47) Premaxilla(e): present as a pair of elements (0); fused as a median element (1). (Modified from X30; XM36; XMZ36; XA43)

(48) Mobile premaxilla: absent (0); present (1). (Modified from GS24; XM37; XMZ37; XA44)

(49) Premaxilla immovably attached to braincase by means of a deep nasal process tightly sutured to frontals: absent (0); present (1). (G6; XW30; XGC45; XM38; XMZ38; XA45)

(50) Number of marginal teeth on premaxilla: three or more (0); one or two (1). (XA46)

(51) Foramen for olfactory nerve on premaxilla: absent (0); present (1). (Modified from G8; XW20; XGC46; XM39; XMZ39; XA47)

(52) Maxilla/preopercle contact: present (0); absent (1). (C15; XM40; XMZ40; XA48)

(53) Ventral portion of preopercle anteriorly extended, contacting maxilla anteriorly: absent (0); present (1). (Modified from XA49)

(54) Supramaxilla: absent (0); present (1). (Modified from GS22; XM41; XMZ41; XA50)

(55) Number of supramaxilla: single (0); two (1). (XZ28; XM42; XMZ42; XA51)

(56) Maxilla: present (0); absent (1). (XM43; XMZ43; XA52)

(57) Mobile maxilla in cheek: absent (0); present (1). (C16; XM44; XMZ44; XA53)

(58) Suborbital/maxilla contact: present (0); absent (1). (Modified from CA88; XGC56; XM45; XMZ45; XA54)

(59) Expanded dorsal lamina of maxilla: present (0); absent (1). (Modified from CA18; XA55)

(60) Depth of dorsal lamina of maxilla: no smaller than orbital length (0); smaller than orbital length (1). (XA56)

(61) Posterior margin of maxilla: slightly convex or straight (0); concave with a posterior maxillary notch (1). (GB62; XW46; XGC57; XM46; XMZ46; XA57)

(62) Oral margin of maxilla: concave or nearly straight (0); convex (1). (XA58)

(63) Posterior end of maxilla relative to orbit: well behind orbit (0); ending below (or nearly below) posterior orbital margin (1); ending nearly below orbital center or even anteriorly located (2).

(XA59)

(64) Teeth on maxilla: present (0); much reduced or lost (1). (Modified from LZ11; XM47; XMZ47; XA60)

(65) Distribution of teeth on maxilla: most of oral margin of maxilla (0); only anterior portion of oral margin of maxilla (1). (XA61)

(66) Extraordinarily long, fang-like teeth on both jaws: absent (0); present (1). (XA62)

(67) Molariform teeth on coronoid(s), prearticular and pterygoids: absent (0); present (1).

(68) Teeth on dentary: present (0); absent (1). (XM48; XMZ48; XA63)

(69) Quadratomandibular articulation: well behind orbit or below posterior orbit margin (0); nearly below the orbital center or even anteriorly located (1). (Modified from LZ2; X56; XGC58; XM49; XMZ49; XA64)

(70) Coronoid process: absent (0); present (1). (GS17; XGC23; XM50; XMZ50; XA65)

(71) Supra-angular bone in lower jaw: present (0); absent (1). (A67; XM51; XMZ51; XA66)

Palatoquadrate, Hyoid Arch, and Operculo-gular Series

(72) Vomers in adults: paired (0); fused (1). (XZS32; XM22; XMZ22; XA67)

(73) Suspensorium angle: acute (0); nearly vertical (1). (Modified from GSM29; XGC24; XM52; XMZ52; XA68)

(74) Hyomandibula with canal for hyoid branch of nerve VII: absent (0); present (1). (Modified from C22; XGC25; XM53; XMZ53; XA69)

(75) Dermohyal: present (0); absent (1). (Modified from GSM24; XM54; XMZ54; XA70)

(76) Postspiracle: absent (0); present (1). (XM55; XMZ55; XA71)

(77) Quadratojugal: plate-like (0); splint-like (1); much reduced or lost (2). (Modified from GSM26; C20; XGC26; XM56; XMZ56; XA72)

Remarks: The codings for *Cleithrolepidina* and *Cleithrolepis* are revised from ‘?’ to ‘0’ based on the descriptions of Hutchinson (1973b).

(78) Symplectic: absent (0); present (1). (Modified GSM13; XGC27; XM57; XMZ57; XA73)

(79) Symplectic involvement of jaw joint: absent (0); present (1). (GB61; G49; XW37; XGC28; XM58; XMZ58; XA74)

(80) Elongated posteroventral process of quadrate: absent (0); present (1). (Modified from GML24; XGC29; XM59; XMZ59; XA75)

(81) Number of hypobranchials: three (0); four (1). (G99; XW42; XGC60; XM60; XMZ60; XA76)

(82) Uncinate processes on epibranchials: absent (0); present (1). (C25; XGC30; XM61; XMZ61; XA77)

(83) Interopercle: absent (0); present (1). (GS18; XGC31; XM62; XMZ62; XA78)

(84) Two or more preopercular elements on each side of skull: absent (0), present (1). (XZ39; XM63; XMZ63; XA79)

(85) Preopercle: present (0); absent (1). (XA80)

(86) Shape of preopercle: boomerang-shaped or irregular (0); crescent-shaped or L-shaped (1). (Modified from XZ40; XM64; XMZ64; XA81)

(87) Ventral end of preopercle located well above posterior end of oral margin of maxilla (when the ventral part of preopercle contacts maxilla): present (0); absent (1). (Modified from XA82)

(88) Opercle: present (0); absent (1). (XA83)

(89) Size of opercle: significantly larger than subopercle (0); nearly equal to or smaller than subopercle (1); much reduced (2). (Modified from XW43; XGC65; XM65; XMZ65; XA84)

(90) Suture between opercle and subopercle: slightly inclined or horizontal (0); greatly inclined (1). (XM66; XMZ66; XA85)

(91) Prominent anterodorsal process of subopercle: absent (0); present (1).

(92) Prominent anteroventral extension of subopercle: absent (0); present (1). (XA86)

(93) Number of branchiostegal rays: ten or more (0); seven to nine (1); four to six (2) two or three (3); single (4). (Modified from XA87)

(94) Gular bone(s): present (0); absent (1). (Modified from C11; XGC58; XM67; XMZ67; XA88)

Vertebrate and Caudal Skeleton

(95) Solid vertebral centra of adult-sized individuals: absent (0); present (1). (Modified from GB4; XGC68; XM68; XMZ68; XA89)

(96) Epipleural intermuscular bones: absent (0); present (1). (A103; XM69; XMZ69; XA90)

(97) Uroneural: absent (0); present (1). (XW56; XM70; XMZ70; XA91)

(98) Division of hypurals into dorsal and ventral groups (a gap between hypurals 2 and 3): absent (0); present (1). (XZS69; XM71; XMZ71; XA92)

Girdles and Fins

(99) Posttemporal: contacting extrascapular posteriorly (0); contacting extrascapular posterolaterally and separating this bone from contact with its counterpart (1); contacting extrascapular medially, and being

incorporated into the skull roof (2); lost (3). (Modified from X27; XGC9; XM72; XMZ72; XA93)

(100) Width of posttemporals: broad, nearly as wide as extrascapular (0); relatively narrow, about half width of extrascapular series (1). (XA94)

(101) Clavicle: present as a broad plate (0); much reduced or lost (1). (Modified from GML37; XGC39; XM73; XMZ73; XA95)

(102) Supracleithrum relative to posterior margin of opercle in depth: supracleithrum nearly as deep as posterior margin of opercle (0); supracleithrum shorter than posterior margin of opercle (1); supracleithrum well deeper than posterior margin of opercle (2).

(103) Pectoral fins enlarged as wings: absent (0); present (1). (X66; XM74; XMZ74; XA96)

(104) Pelvic fins enlarged as auxiliary wings: absent (0); present (1). (X70; XM75; XMZ75; XA97)

(105) Number of dorsal and anal fin rays relative to radials: rays more numerous than radials (0); rays and radials nearly equal in number (1). (GSM31; XGC34; XM76; XMZ76; XA98)

Remarks: In *Cleithrolepis granulate*, the rays are more numerous than the radials in both the dorsal (19 radials corresponding to 27 rays) and anal (9 radials corresponding to 19 rays) fins (Wade, 1935). The similar condition is also present in *Polzbergia brochatus*, in which, the rays are also more numerous than the radials in the dorsal fin (Griffith, 1977).

(106) Dorsal and anal fin rays: segmented throughout the length (0); segmented distally (1). (XG62; XGC35; XM77; XMZ77; XA99)

(107) Origin of dorsal fin: nearly opposite to the origin of anal fin, well posterior to the origins of pelvic fins (0); between origins of anal and pelvic fins (1); anterior to origins of pelvic fins (2). (XA100)

(108) Caudal fin rays: terminate at caudal extremity of body axis (0); extend beyond termination of body axis (1). (Modified from GSM32; XGC36; XM78; XMZ78; XA101)

(109) Epaxial procurrent rays in dorsal lobe of caudal fin: absent (0); present (1). (Modified from LZ19; XA102)

(110) Number of epaxial procurrent rays in dorsal lobe of caudal fin: four or more (0); one to three (1).

(111) Number of principal caudal fin rays: 25 or more (0); 24 or less (1). (XA104)

(112) Principal rays of caudal fin ornamented with rounded ganoid tubercles: absent (0); present (1).

(113) Dense lepidotrichial segments of pectoral fin rays between innermost principle pectoral fin ray and body: absent (0); present (1). (X71; XGC37; XM79; XMZ79; XA105)

(114) Dense brush-like rays proximally articulating several stout segments at posterior portion of male

anal fin: absent (0); present (1). (Modified from XZS74; XM80; XMZ80; XA106)

(115) Modification of male anal fin into unsegmented rays with tiny hooklets along anterior margin of longest leading ray: absent (0); present (1). (XM81; XMZ81; XA107)

(116) Enlarged and posteriorly extended lateral scutes associate with anal fin: absent (0); present (1). (Modified from XM82; XMZ82; XA108)

(117) Anal fin relative to dorsal fin in size: anal fin smaller than or equal to dorsal fin (0); anal fin larger than dorsal fin (1). (XM83; XMZ83; XA109)

(118) Caudal fin: forked, lower lobe slightly shorter than or largely equal to upper lobe (0); forked, lower lobe longer than upper lobe (1); unforked (2). (Modified from X76; XGC74; XM84; XMZ84; XA110)

(119) Fringing fulcra on caudal fin: present (0); absent (1). (XM85; XMZ85; XA111)

(120) Fringing fulcra on pectoral fins: present (0); absent (1). (XM86; XMZ86; XA112)

Body shape and scales

(121) An apparent dorsal hump between head and dorsal fin: absent (0); present (1). (XW50; XA113)

(122) Scales: present (0); absent (1). (Modified from XGF67; XM87; XMZ87; XA114)

(123) Anterior flank scales: present (0); much reduced or absent (1). (Modified from XZS83; XM89; XMZ89; XA115)

(124) Lateral line scales: the lateral line scales as deep as or slightly deeper than those scales above and below (0); greatly deepened, 30% or more of the greatest depth of the body (1). (Modified from XZS83; XM89; XMZ89; XA116)

(125) Two horizontal rows of scales notably deepened in anterior flank region (lateral line scales notably deepened and nearly equal to the scales ventral to them): absent (0); present (1).

(126) Greatly deepened anterior flank scales corresponding to two or three horizontal rows of relatively shorter scales posteriorly: absent (0); present (1).

(127) Rhombic scales in anterior flank region: ornamented with ganoid ridges and tubercles (0); nearly smooth (1).

(128) Type of scales: ganoid of lepisosteoid type (0); elasmoid of amioid type (1); elasmoid of cycloid type (2). (A156; X80; XGC71; XM90; XMZ90; XA117)

(129) A posteriorly directed spine on dorsal ridge scale anterior to dorsal fin: absent (0); present (1). (GML36; LZ22; XW58; XA118)

(130) Posteriorly inclined scales in pectoral region: absent (0); present (1). (LZ24; XM92; XMZ92; XA119)

4. Data matrix

Moythomasia
00000000000-0-0----00000000000-00000000-000--000000000-00-0000000000000001000000000000
00000000000000000000000-00000000000000000000
Pteronisculus
00000000000-0-0----000000000100010000010010--2-00-0000-0001000000000000001000000000000
00000000000000000000100-00000000000000000000
Boreosomus_piveteaui
00000000000-0-0----000000010100000100010010--2-00-0000-00101000000000000000?000000000
02000000000000010000200-00000000000000000000
Acipenser
21020000111-0-0001000010110010?0?-10000---1002-00----0-1----------0-001-0010000000001-
-1--00310000200-0000100-0000000001000----000
Chondrosteus_acipenseroides
2?010000001-0-0???0?????1?0?1????-10000---0--2-00----0-1----------0-001-00100000??001-
-0200011000001000000200-0000000001001----000
Peipiaosteus_pani
2?01?0001?1-0-0???0?????1????????-10000---0--2-00----0-1----------0-001-00100000??001-
-020002100002?000000100-0000000001001----000
Australosomus
000000101210000???001101010010??00?0-010010--???0?0000-0010100000000000001000000010000
00000010000000010000011000000010110002001000
Brookvalia_gracilis
010020101210000???0?????????101000100010011002-00--000-000010000000000??0?002?????0000
0010004?????00020000010-00000000000000000000
Daedalichthys_higginsi
010010001210000???0?????????101000100010011002-00--000-00?010000000000??0?002?????0000
0010003?????00020000010-00000000000000000000
Redfieldius_gracilis
010010101010010???0?????????101000100010011002-00--000-001010000000000??0?002?????0000
0010004?????00020000011000000000000000001000
Helichthys_browni
010010101210010???0?????????101000100010011002-00--000-001010000000000??0?002?????0000
0010004?????00020000011000000000000000001000
Platysiagum
000100010210000???0?????????10000011000-010--10?000000-001010000000000??1?00??????0000
001100100???00000000110-0000000000000000(01)000
Helmolepis_cyphognathus
000100010010000???0?????????10000001000-010--??????000-001010000000000??1?00??????0000
000100100???00000000110-00000000000000001000
Cleithrolepidina

000100000211100???0?????????100000110111?0120??00-0000-00101001100010O??1?000?????0000
001000??0???3-?100?011??000000000001000000001
Cleithrolepis
000?00000011100???0?????????10000011011100120100O-0000-001010011000(01)00??1?000?????0
0000010000?0???3-??00001110000000000?1000000001
Polzbergia
?00?000??21?000???0?????????1???001101????120100000O?0-001010?1100000???1?00??????0?00
?0000???????3-0100000110000000000?0000000000
Plesiofuro
000100000211000???0?????????1000001001110012010000000O-0010100000000000?1?002?????0000
001000200???00?00011110-10000000000000001000
Teffichthys_
000100000211000---01110?10001000001001110012010000000O-00101000000100O??11002??00?0000
001000200???01?00011111010000000000000000001000
Meidiichthys_browni
000100000211000???0?????????1000001001110012010000000O-0010100000O?000??1?002??00?0000
00100020????00000011110-10000000000000001000
Perleidus_altolepis
000100000211000???0?????????1000001001110012010000000O-00101000000100O??1?002?????0000
00100020????01?00011111010000000000000000001000
Peltoperleidus_ducanensis
0001100?0210000???0?????????100000100111001?010000001O-001010010100000??1?002?????0000
00100130????01?00011110-100000000000000101000
Peltoperleidus_macrodontus
0001-2-00?1-000???0?????????1000001001110012010001001O-0010100101000O???1?002?????0000
0010013?????01?00011110-100000000000000101000
Pseudobeaconia_bracaccinii
010100000010000???0?????????10000011010-00110100000010-001010010100000??1?002?????0000
00100130????0100001111111000000000?0000001010
Dipteronotus_olgiatii
000100000211000???0?????????100000100111001OO??????010-00101001010?000??1???2?????0000
?01001??????0??000?1111?100000000?1000001010
Louwoichthys_illus
00010(02)000210000???0?????????1000001001110012010001001O-0010100101100000?1?002?????0
000001001300???01000011111010000000000000000001000
Ctenognathichthys
000100000210000???0?????????1000001001110012010001001O-00100001011000O??1?002?????0000
0010013?????01?000111110000000000000000001000
Luopingichthys_bergi
000100000210000???0?????????1000001001110012010001001O-0010100101100000?1?002?????0000
001001300???01000011111000000000000000000001000
Colobodus_giganteus
010000000211000???0?????????1000??11010-001211000000000-00-010000001000??1?002?????0000
0000100?????0??000?111110?000000000000000000

Colobodus_bassanii
010000000211000???0?????????10000011010-001201000?0000-00-010000010000?11002?????0000
000010000???01?000111111010000000000000000000
Colobodus_baii
010000000211000???0?????????1000001?0?0-001?1100000000-00-010000010000?11002?????0000
0000?0??????01?000?1111001000000000000000000
Crenilepis_sandbergeri
010000000211000???0?????????10000011010-00121100000000-00-010000010000?11002?????0000
000010000???01?000111110010000000000000000000
Feroxichthys_yunnanensis
00000100021-000???0?????????1000-111010-00121110000000-00-010000101000??1?002?????0000
00001010????01?000?11110?1000000000000001000
Feroxichthys_panzhouensis
???????????????????????????????-111010-00121??????000-00-0001001010000?1?002?????0000
00001010???????000?11110010000?0001000000000
Luganoia_lepidosteoides
10210100001-010???0?????????1000-1100110001213100000000-00001002010101010??11002?????0100
-01001??????01?100112110100000021100010110000
Luganoia_fortuna
10210100011-010???0?????????1000-1100110001??310000000-0000100201?0010??1?002?????0000
-01001??????01?1001121101000000211000101100000
Fuyuanperleidus_dengi
00010100021-000???0?????????1000011001110012010000?0000-0010100001010000?1?002?????0000
00000020????01?100111111000000000110001011000
Altisolepis_bellipinnis
0001000002100000???0?????????10000010011100120??????000-0010100000000000??11?02?????0000
00000020????01?100?1111010000010110001001000
Peltopleurus_nitidus
00011(02)000110010???0?????????10000010011100120100000000-00101000010?000??1?012?????0
00010000030????01010011111010010110000001001000
Peltopleurus_tyrannos
00011(02)000010010???0?????????10000010011100110100000000-00101000010100 0??1?012?????0
00010000020????0??100?1111010010110000001001000
Peltopleurus_rugosus
0001100001100 10???0?????????10000010011100110??00?0000-00101000010?000??1?0?2?????0000
10000020????01?10011111010010100010001001000
Wushaichthys
001111-0021-010???0?????????10100010011000120100000000-00101000010?000??1?012?????0000
1000002000??1101001111101001010000000100100 0
Peripeltopleurus
001111-0021-010???0?????????10100010011000120100000000-00101000010?000??1?012?????0000
100000200???11?100111110100101010000001001000
Thoracopterus
001111-1121-010???0?????????10100010011???100100000000-00101000010?000??1?????????0000

100100000??011?111111110101???01110001001000
Potanichthys
001111-0121-010???0?????1?0010100010011000120110000000-00101000000001000??1??12?000?0000
100100?00?0011?1111111101011010111001----000
Gigantopterus
001111-??21-0?0???0?????????10?0001?01??00120?????0000-001010?00?0?000??11????????0000
?00100??0??01??111111110?01?0??111001----000
Thoracopterus_magnificus
001111-01?1-010???0?????????10?00010011?00???110000000-00101000000?000??1???2?00??0000
100100000?0011?111111110101?0?0111011-----00
Thoracopterus_martinisi
0011-2-01?1-010???0?????????10100010011?00???110?0?000-00101000000?000??1??12?00??0000
100100??0?0011?111111110?01?0?0111011-----00
Venusichthys
0001000002100000???0?????????100000100110110--100000000-001010110100011??1?102?????0100
100000310???010100111110100000000000001000000
Habroichthys
1001-2-0?21-010???0?????????10?00011010-??0--1000??1-0-011010021-0010???1?102??0??0001
-000004?0???01?1011111101000100100011100100 0
Caturus
10010001121100111100?????1?0010110010101011121100 1011-100111-10000000100111021101?1001
-0001000000000?00011210-0000000000000000-100
Amia
1001000112100011100101011000101100101 00-110--1001011-100111-100000001001110211011 1001
-00010000000001000112 10-1000000211000000-100
Lepisosteus
1001000112100011110001111000111100100110111101001011-100---------0001100110011001 10001
-0001031000001100011010-10000012000000001000
Semionotus
10010001121000111 10?????1?001111001000101112 01001011-100111-0020100011001110110011 1001
-0001011000001110011110-1000000000000000 1010
Kyphosichthys_grandei
10010001121001 1???0?????????1111001001101112010010?1-100111-0020000001 0?1?101?????1001
-00010100???00110011110-10000000001000000010
Leptolepis_
1001000112100000000111101101110000010011011111010 10001-110111-01000000011111102101011001
-000000011110011001111 0-1000000000000000-200
Elops
1001000112110000101 1?10111101000001001 0-111001010001-110111-01000000011111102101011001
-0000000111100100011210-1000000011000000-200

5. References to Supplementary Information

Arratia G. 1999. The monophyly of Teleostei and stem-group teleosts. Consensus and disagreements. In: Arratia G, Schultze H-P, eds. *Mesozoic fishes 2 – systematics and fossil record*. München: Verlag Dr. Friedrich Pfeil, 265–334.

Arratia G. 2013. Morphology, taxonomy, and phylogeny of Triassic pholidophorid fishes (Actinopterygii, Teleostei). *Society of Vertebrate Paleontology Memoir (Supplement to Journal of Vertebrate Paleontology)* **13:** 1–138.

Brough J. 1931. The Triassic fishes of the Karroo System and some general considerations on the bony fishes of the Triassic period. *Proceedings of the Zoological Society* **1931:** 235–296.

Bürgin T. 1992. Basal ray-finned fishes (Osteichthyes; Actinopterygii) from the Middle Triassic of Monte San Giorgio (Canton Tessin, Switzerland). *Schweizerische Palaontologische Abhandlungen* **114:** 1–164.

Cartanyà J, Fortuny J, Bolet A, Mutter RJ. 2015. *Colobodus giganteus* (Beltan, 1972) comb. nov. from the Upper Muschelkalk facies of Catalonia (NE Iberian Peninsula). *Neues Jahrbuch für Geologie und Paläontologie, Abhandlungen* **278:** 323–333.

Cavin L. 2010. Diversity of Mesozoic semionotiform fishes and the origin of gars (Lepisosteidae). *Naturwissenschaften* **97:** 1035–1040.

Cloutier R, Arratia G. 2004. Early diversification of actinopterygians; In: Arratia G, Wilson MVH, Cloutier R, eds. *Recent Advances in the Origin and Early Radiation of Vertebrates*. München: Verlag Dr. Friedrich Pfeil, 217–270.

Coates MI. 1999. Endocranial preservation of a Carboniferous actinopterygian from Lancashire, UK, and the interrelationships of primitive actinopterygians. *Philosophical Transactions of the Royal Society of London B* **354:** 435–462.

Forey PL. 1973. A revision of the elopiform fishes, fossil and Recent. *Bulletin of the British Museum (Natural History), Geology* **10(Suppl):** 1–222.

Gardiner BG. 1984. The relationships of the palaeoniscid fishes, a review based on new specimens of *Mimia* and *Moythomasia* from the Upper Devonian of Western Australia. *Bulletin of the British Museum (Natural History), Geology* **37:** 173-428.

Gardiner BG, Maisey JG, Littlewood DTJ. 1996. Interrelationships of basal neopterygians. In: Stiassney MLJ, Parenti LR, Johnson GD, eds. *Interrelationships of Fishes*. San Diego: Academic Press, 117–146.

Gardiner BG, Schaeffer B. 1989. Interrelationships of lower actinopterygian fishes. *Zoological Journal of the Linnean Society* **97:**135–187.

Gardiner BG, Schaeffer B, Masserie JA. 2005. A review of the lower actinopterygian phylogeny. *Zoological Journal of the Linnean Society* **144:** 511–525.

Geng B-H, Jin F, Wu F-X, Wang Q. 2012. New perleidid fishes from the Middle Triassic strata of Yunnan Province. *Geological Bulletin of China* **31:** 915–927.

Grande L, Bemis WE. 1998. A comprehensive phylogenetic study of amiid fishes (Amiidae) based on comparative skeletal anatomy: An empirical search for interconnected patterns of natural history. *Society of Vertebrate Paleontology Memoir (supplement to Journal of Vertebrate Paleontology)* **4:**1–690.

Grande L. 2010. An empirical synthetic pattern study of gars (Lepisosteiformes) and closely related species, based mostly on skeletal anatomy. The resurrection of Holostei. *Copeia* **10 (Supplement):** 1–871.

Griffith J. 1977. The Upper Triassic fishes from Polzberg bei Lunz, Austria. *Zoological Journal of the Linnean Society* **60:** 1–93.

Hilton EJ, Forey PL. 2009. Redescription of †*Chondrosteus acipenseroides* Egerton, 1858 (Acipenseriformes, †Chondrosteidae) from the lower Lias of Lyme Regis (Dorset, England), with comments on the early evolution of sturgeons and paddlefishes. *Journal of Systematic Palaeontology* **7:** 427–453.

Hilton EJ, Grande L, William EB. 2011. Skeletal anatomy of the shortnose sturgeon, *Acipenser brevirostrum* Lesueur, 1818, and the systematics of sturgeons (Acipenseriformes, Acipenseridae). *Fieldiana–Life Earth Sciences* **3:** 1–168

Hutchinson P. 1973a. *Pseudobeaconia,* a perleidiform fish from the Triassic Santa Clara Formation, Argentina. *Breviora* **398:**1–24.

Hutchinson P. 1973b. A revision of the redfieldiiform and perleidiform fishes from the Triassic of Bekker's Kraal (South Africa) and Brookvale (New South Wales). *Bulletin of the British Museum of Natural History (Geology)* **22:** 235–354.

Lehman JP. 1952. Etude complémentaire des possions de l'Eotrias de Madagascar. *Kungliga Svenska Vetenskapsakademiens Hangdlingar* **2:** 1–201.

Lehman JP. 1979. Note sur les Poissons du Trias de Lunz. I. *Thoracopterus* Bronn. *Annalen des Naturhistorischen Museums in Wien* **82:** 53–66

Lombardo C. 2001. Actinopterygians from the Middle Triassic of northern Italy and Canton Ticino (Switzerland): anatomical descriptions and nomenclatural problems. *Rivista Italiana di Paleontologia e Stratigrafia* **107:** 345–369.

López-Arbarello A, Zavattieri AM. 2008. Systematic revision of *Pseudobeaconia* Bordas, 1944, and *Mendocinichthys* Whitley, 1953 (Actinopterygii: 'Perleidiformes') from the Triassic of Argentina. *Palaeontology* **51:** 1025–1052.

López-Arbarello A. 2012. Phylogenetic interrelationships of ginglymodian fishes (Actinopterygii: Neopterygii). *PLoS ONE* **7:** e39370.

Marramà G, Lombardo C, Tintori A, Carnevale G. 2017. Redescription of '*Perleidus*' (Osteichthyes, Actinopterygii) from the Early Triassic of northwestern Madagascar. *Rivista Italiana di Paleontologia e Stratigrafia* **123:** 219–242.

Mutter RJ. 2002. Revision of the Triassic family Colobodontidae sensu Andersson, 1916 (emended) whith a tentative assessment of perleidiform interrelationships (Actinopterygii: Perleidiformes). Unpublished Ph.D. thesis, Universität Zürich, Switzerland.

Mutter RI. 2004. The "perleidiform" family colobodontidae: A review. In Arratia G, Tintori A, eds. *Mesozoic Fishes 3 – Systematics, Paleoenvironments and Biodiversity.* München: Verlag Dr. Friedrich Pfeil, 197–208.

Neuman AG, Mutter RJ. 2005. *Helmolepis cyphognathus,* sp. nov., a new platysiagid actinopterygian from the lower Triassic Sulphur Mountain Formation (British Columbia, Canada). *Canadian Journal of Earth Sciences* **42:** 25–36.

Nielsen E. 1942. Studies on Triassic fishes from East Greenland. I. *Glaucolepis* and *Boreosomus*. *Meddelser øm Grønland* **138:** 1–403.

Nielsen E. 1949. Studies on Triassic fishes from East Greenland. II. *Australosomus* and *Birgeria*. Meddelser øm Grønland **146:** 1–309.

Olsen PE, McCune AR. 1991. Morphology of the *Semionotus elegans* species group from the Early Jurassic part of the Newark Supergroup of eastern North America, with comments on the family Semionotidae (Pisces: Neopterygii). *Journal of Vertebrate Paleontology* **11:** 269–292.

Patterson C. 1975. The braincase of pholidophorid and leptolepid fishes, with a review of the actinopterygian braincase. *Philosophical Transactions of the Royal Society of London, Series B* **269:** 275–579.

Pinna MCC. 1996. Teleostean monophyly. In: Stiassny MLJ, Parenti LR, Johnson GD eds. *Interrelationships of Fishes*. San Diego: Academic Press, 147–162.

Schaeffer B, McDonald NG. 1978. Redfieldiid fishes from the Triassic-Liassic Newark Supergroup of eastern North America. *Bulletin of the American Museum of Natural History* **159:** 129–173.

Sun Z-Y, Lombardo C, Tintori A, Jiang D-Y, Hao W-C, Sun Y-L, Lin H-Q. 2012. *Fuyuanperleidus dengi* Geng et al. 2012 (Osteichthyes, Actinopterygii) from the Middle Triassic of Yunnan Province,

South China. *Rivista Italiana di Paleontologia e Stratigrafia* **118:** 359–373.
Sun Z-Y, Tintori A, Jiang D-Y, **Lombardo C**, **Rusconi M, Hao W-C, Sun Y-L. 2009.** A new perleidiform (Actinopterygii, Osteichthyes) from the Middle Anisian (Middle Triassic) of Yunnan, South China. *Acta Geologica Sinica* **83:** 460–470.
Sun Z-Y, Tintori A, Lombardo C, **Jiang D-Y**, **Hao W-C, Sun Y-L, Wu F-X, Rusconi M. 2008.** A new species of the genus *Colobodus* Agassiz, 1844 (Osteichthyes, Actinopterygii) from the Pelsonian (Anisian, Middle Triassic) of Guizhou, South China. *Rivista Italiana di Paleontologia e Stratigrafia* **114:** 363–376.
Tintori A. 1990. *Dipteronotus olgiatii* n. sp. (Actinopterygii, Perleidiformes) from the Kalkschieferzone of Ca' del Frate (N. Italy). *Atti Ticinensi di Scienze della Terra* **33:** 191–197.
Tintori A, Sassi D. 1992. *Thoracopterus* Bronn (Osteichthyes: Actinopterygii): a gliding fish from the Upper Triassic of Europe. *Journal of Vertebrate Paleontology* **12:** 265–283.
Wade RT. 1935. The Triassic Fishes of Brookvale, New South Wales. British Museum (Natural History), London, 110 pp.
Wen W, Hu S-X, Zhang Q-Y, Benton MJ, Kriwet J, Chen Z-Q, Zhou C-Y, Xie T, Huang J-Y. **2019.** A new species of *Platysiagum* from the Luoping Biota (Anisian, Middle Triassic, Yunnan, South China) reveals the relationship between Platysiagidae and Neopterygii. *Geological Magazine* **156:** 669–682.
Xu G-H. 2020a. A new stem-neopterygian fish from the Middle Triassic (Anisian) of Yunnan, China, with a reassessment of the relationships of early neopterygian clades. *Zoological Journal of the Linnean Society*. DOI: 10.1093/zoolinnean/zlaa053.
Xu G-H. 2020b. A new species of *Luganoia* (Luganoiidae, Neopterygii) from the Middle Triassic Xingyi Biota, Guizhou, China. *Vertebrata PalAsiatica* **58:** 267–282.
Xu G-H. 2020c. *Feroxichthys yunnanensis* gen. et sp. nov. (Colobodontidae, Neopterygii), a large durophagous predator from the Middle Triassic (Anisian) Luoping Biota, eastern Yunnan, China. *PeerJ* **8:** e10229.
Xu G-H, Gao K-Q. 2011. A new scanilepiform from the Lower Triassic of northern Gansu Province, China, and phylogenetic relationships of non-teleostean Actinopterygii. *Zoological Journal of the Linnean Society* **161:** 595–612.
Xu G-H, Gao K-Q, Coates MI. 2015a. Taxonomic revision of *Plesiofuro mingshuica* from the Lower Triassic of northern Gansu, China, and the relationships of early neopterygian clades. *Journal of Vertebrate Paleontology* **35:** e1001515.
Xu G-H, Gao K-Q, Finarelli JA. 2014. A revision of the Middle Triassic scanilepiform fish *Fukangichthys longidorsalis* from Xinjiang, China, with comments on the phylogeny of the Actinopteri. *Journal of Vertebrate Paleontology* **34:** 747–759.
Xu G-H, Ma X-Y. 2016. A Middle Triassic stem-neopterygian fish from China sheds new light on the peltopleuriform phylogeny and internal fertilization. *Science Bulletin* **61:** 1766–1774.
Xu G-H, Ma X-Y, Zhao L-J. 2018. A large peltopleurid fish from the Middle Triassic (Ladinian) of Yunnan and Guizhou, China. *Vertebrata PalAsiatica* **56:** 106–120.
Xu G-H, Wu F-X. 2012. A deep-bodied ginglymodian fish from the Middle Triassic of eastern Yunnan Province, China, and the phylogeny of lower neopterygians. *Chinese Science Bulletin* **57:**111−118.
Xu G-H, Zhao L-J. 2016. A Middle Triassic stem-neopterygian fish from China shows remarkable secondary sexual characteristics. *Science Bulletin* **61:** 338–344.
Xu G-H, Zhao L-J, Gao K-Q, Wu F-X. 2012. A new stem-neopterygian fish from the Middle Triassic of China shows the earliest over-water gliding strategy of the vertebrates. *Proceedings of the Royal Society B* **280:** 20122261.
Xu G-H, Zhao L-J, Shen C-C. 2015b. A Middle Triassic thoracopterid from China highlights the evolutionary origin of over-water gliding in early ray-finned fishes. *Biology Letters* **11:** 20140960. Doi:10.1098/rsbl.2014.0960.
Zhou Z-H. 1992. Review on *Peipiaosteus* based on new material of *P. pani*. *Vertebrata PalAsiatica* **30:** 85–101.
